# Indoleamine 2,3-Dioxygenase Deletion to Modulate Kynurenine Pathway and to Prevent Brain Injury after Cardiac Arrest in Mice

**DOI:** 10.1097/ALN.0000000000004713

**Published:** 2023-07-24

**Authors:** Aurora Magliocca, Carlo Perego, Francesca Motta, Giulia Merigo, Edoardo Micotti, Davide Olivari, Francesca Fumagalli, Jacopo Lucchetti, Marco Gobbi, Alessandra Mandelli, Roberto Furlan, Markus B. Skrifvars, Roberto Latini, Giacomo Bellani, Fumito Ichinose, Giuseppe Ristagno

**Affiliations:** 1 Department of Pathophysiology and Transplants, University of Milan, Milan, Italy; and Department of Cardiovascular Medicine, Istituto di Ricerche Farmacologiche Mario Negri IRCCS, Milan, Italy.; 2 Department of Cardiovascular Medicine, Istituto di Ricerche Farmacologiche Mario Negri IRCCS, Milan, Italy.; 3 Department of Cardiovascular Medicine, Istituto di Ricerche Farmacologiche Mario Negri IRCCS, Milan, Italy.; 4 Department of Anesthesia, Critical Care and Emergency, Fondazione IRCCS Ca' Granda Ospedale Maggiore Policlinico, Milan, Italy.; 5 Department of Neuroscience, Istituto di Ricerche Farmacologiche Mario Negri IRCCS, Milan, Italy.; 6 Department of Cardiovascular Medicine, Istituto di Ricerche Farmacologiche Mario Negri IRCCS, Milan, Italy.; 7 Department of Cardiovascular Medicine, Istituto di Ricerche Farmacologiche Mario Negri IRCCS, Milan, Italy.; 8 Department of Biochemistry and Molecular Pharmacology, Istituto di Ricerche Farmacologiche Mario Negri IRCCS, Milan, Italy.; 9 Department of Biochemistry and Molecular Pharmacology, Istituto di Ricerche Farmacologiche Mario Negri IRCCS, Milan, Italy.; 10 Clinical Neuroimmunology Unit, Division of Neuroscience, Institute of Experimental Neurology–INSpe, San Raffaele Scientific Institute, Milan, Italy.; 11 Clinical Neuroimmunology Unit, Division of Neuroscience, Institute of Experimental Neurology–INSpe, San Raffaele Scientific Institute, Milan, Italy.; 12 Department of Emergency Care and Services, Helsinki University Hospital and University of Helsinki, Finland.; 13 Department of Cardiovascular Medicine, Istituto di Ricerche Farmacologiche Mario Negri IRCCS, Milan, Italy.; 14 Centre for Medical Sciences−CISMed, University of Trento, Italy; and Department of Anesthesia and Intensive Care, Santa Chiara Hospital, Trento, Italy.; 15 Anesthesia Center for Critical Care Research of the Department of Anesthesia, Critical Care and Pain Medicine, Massachusetts General Hospital, Boston, Massachusetts; and Harvard Medical School, Boston, Massachusetts.; 16 Department of Pathophysiology and Transplants, University of Milan, Milan, Italy; and Department of Anesthesia, Critical Care and Emergency, Fondazione IRCCS Ca’ Granda−Ospedale Maggiore Policlinico, Milan, Italy.

## Abstract

**Background:**

The catabolism of the essential amino acid tryptophan to kynurenine is emerging as a potential key pathway involved in post–cardiac arrest brain injury. The aim of this study was to evaluate the effects of the modulation of kynurenine pathway on cardiac arrest outcome through genetic deletion of the rate-limiting enzyme of the pathway, indoleamine 2,3-dioxygenase.

**Methods:**

Wild-type and indoleamine 2,3-dioxygenase–deleted (IDO^−/−^) mice were subjected to 8-min cardiac arrest. Survival, neurologic outcome, and locomotor activity were evaluated after resuscitation. Brain magnetic resonance imaging with diffusion tensor and diffusion-weighted imaging sequences was performed, together with microglia and macrophage activation and neurofilament light chain measurements.

**Results:**

IDO^−/−^ mice showed higher survival compared to wild-type mice (IDO^−/−^ 11 of 16, wild-type 6 of 16, log-rank *P* = 0.036). Neurologic function was higher in IDO^−/−^ mice than in wild-type mice after cardiac arrest (IDO^−/−^ 9 ± 1, wild-type 7 ± 1, *P* = 0.012, n = 16). Indoleamine 2,3-dioxygenase deletion preserved locomotor function while maintaining physiologic circadian rhythm after cardiac arrest. Brain magnetic resonance imaging with diffusion tensor imaging showed an increase in mean fractional anisotropy in the corpus callosum (IDO^−/−^ 0.68 ± 0.01, wild-type 0.65 ± 0.01, *P* = 0.010, n = 4 to 5) and in the external capsule (IDO^−/−^ 0.47 ± 0.01, wild-type 0.45 ± 0.01, *P* = 0.006, n = 4 to 5) in IDO^−/−^ mice compared with wild-type ones. Increased release of neurofilament light chain was observed in wild-type mice compared to IDO^−/−^ (median concentrations [interquartile range], pg/mL: wild-type 1,138 [678 to 1,384]; IDO^−/−^ 267 [157 to 550]; *P* < 0.001, n = 3 to 4). Brain magnetic resonance imaging with diffusion-weighted imaging revealed restriction of water diffusivity 24 h after cardiac arrest in wild-type mice; indoleamine 2,3-dioxygenase deletion prevented water diffusion abnormalities, which was reverted in IDO^−/−^ mice receiving l-kynurenine (apparent diffusion coefficient, μm^2^/ms: wild-type, 0.48 ± 0.07; IDO^−/−^, 0.59 ± 0.02; IDO^−/−^ and l-kynurenine, 0.47 ± 0.08; *P* = 0.007, n = 6).

**Conclusions:**

The kynurenine pathway represents a novel target to prevent post–cardiac arrest brain injury. The neuroprotective effects of indoleamine 2,3-dioxygenase deletion were associated with preservation of brain white matter microintegrity and with reduction of cerebral cytotoxic edema.

## Abstract 2

Genetic deletion of indoleamine 2,3-dioxygenase, the rate-limiting enzyme of the kynurenine pathway, increased survival and neurologic function after resuscitation from an 8-min-long cardiac arrest in mice. The protective effects of indoleamine 2,3-dioxygenase deletion were associated with preservation of brain white matter integrity, with a decrease in brain neurofilament light chain release, and with a reduction of ischemia-induced brain edema.

**Editor’s Perspective**

**What We Already Know about This Topic**

Activation of the kynurenine pathway, a major tryptophan metabolism pathway in the body, has been shown to be associated with poor outcome after cardiac arrest.

The question of whether kynurenine pathway activation has a causal role in cardiac arrest–related brain injury is incompletely explored.

**What This Article Tells Us That Is New**

Genetic deletion of indoleamine 2,3-dioxygenase, the rate-limiting enzyme of the kynurenine pathway, increased survival and neurologic function after resuscitation from an 8-min-long cardiac arrest in mice.

The protective effects of indoleamine 2,3-dioxygenase deletion were associated with preservation of brain white matter integrity, with a decrease in brain neurofilament light chain release, and with a reduction of ischemia-induced brain edema.

Cardiac arrest is a leading cause of death worldwide.^1^ Although significant improvement has been achieved in cardiopulmonary resuscitation (CPR) interventions and postresuscitation care, cardiac arrest outcomes still remain poor. Indeed, only 10% of out-of-hospital cardiac arrest patients survive to hospital discharge, and more than 80% of these survivors show long-term unfavorable neurologic outcome.^1^ Post–cardiac arrest brain injury accounts for the majority of deaths in patients admitted to intensive care unit after resuscitation and is ultimately the main determinant of functional outcome in surviving patients.^2,3^ The pathophysiological processes of post–cardiac arrest brain injury, starting with cessation of blood flow and culminating in neuronal death, have not been entirely unveiled yet. Identified damaging pathways include excitotoxicity, free radical formation, endothelial dysfunction, disrupted calcium homeostasis, and mitochondrial dysfunction.^4^

The metabolism of the essential amino acid tryptophan to kynurenine activates the so-called kynurenine pathway. Metabolites of the kynurenine pathway have emerged as potential key components involved in the mechanisms responsible for brain injury after cardiac arrest.^5–7^

Tryptophan is metabolized into kynurenine mainly by the enzyme indoleamine 2,3-dioxygenase. Inflammation can induce indoleamine 2,3-dioxygenase expression in a variety of tissues, including in macrophages and dendritic cells *via* interferon-γ or tumor necrosis factor.^8^ Kynurenine has been identified as an endothelium-dependent relaxing factor contributing to the regulation of blood pressure in systemic inflammation.^9^ Downstream metabolites of the kynurenine pathway include the neurotoxic 3-hydroxyanthranilic acid and kynurenic acid, a neuroprotective metabolite acting as an *N*-methyl-d-aspartate antagonist.^10^

In a translational study including small and large animals, subsequently confirmed in patients, it has been observed that the kynurenine pathway was activated early after cardiac arrest.^11^ Thus, plasmatic reduction of tryptophan together with increased levels of kynurenine, kynurenic acid, and 3-hydroxyanthranilic acid was consistently observed in rats and pigs from the first hours after resuscitation up to 3 to 5 days after cardiac arrest. Of particular interest, an early activation of the kynurenine pathway has been highlighted in out-of-hospital cardiac arrest patients, and higher plasmatic levels of kynurenine pathway metabolites predicted poor neurologic outcome at intensive care unit (ICU) discharge and at 12 months after cardiac arrest.^5^

Although kynurenine pathway activation has been associated with poor outcome, its causative role on brain injury and survival after cardiac arrest remains to be elucidated. Recently, our group has reported that pharmacologic pretreatment with an indoleamine 2,3-dioxygenase inhibitor before inducing cardiac arrest in rats improved post–cardiac arrest neurologic function while reducing systemic and cerebral kynurenine pathway activation.^7^ Altogether, this evidence suggests that the inhibition of the rate-limiting enzyme indoleamine 2,3-dioxygenase could represent a novel therapeutic target to reduce post–cardiac arrest brain injury and improve outcome.

The aim of this experimental study was to elucidate the direct impact of kynurenine pathway on outcome of cardiac arrest, using a model of CPR in mice with deletion of indoleamine 2,3-dioxygenase (IDO^−/−^). Specifically, we examined whether knocking out the rate-limiting enzyme for tryptophan catabolism indoleamine 2,3-dioxygenase reduced post–cardiac arrest brain injury while improving survival and neurologic outcome. Furthermore, we assessed whether phenotype rescuing by administration of exogenous l-kynurenine could revert the above benefits, reinforcing its responsible role on brain injury after resuscitation.

## Materials and Methods

This is an experimental randomized animal study, conducted at the Istituto di Ricerche Farmacologiche Mario Negri, Istituto di Ricovero e Cura a Carattere Scientifico (Milan, Italy). The animals were randomized on 1:1 or 1:1:1 basis according to the treatment group in two independent experiments: wild-type *versus* IDO^−/−^ mice were compared for the first set of experiments (n = 16/group), and wild-type *versus* IDO^−/−^ *versus* IDO^−/−^ + l-kynurenine were compared for the second set of experiments on the rescue of the phenotype (n = 6/group). For the first set of experiments, sample size was calculated to test the effect of genetic deletion of indoleamine 2,3-dioxygenase on survival up to 7 days after cardiac arrest. As the mean survival rate at 7 days after cardiac arrest was expected to be 40% in wild-type mice, as shown in a previous study by Minamishima *et al.*,^12^ and 80% in IDO^−/−^ mice (expected effect size on mortality, hazard ratio = 0.25), we anticipate that 16 mice per group were required for the survival study (α = 0.05, β = 0.8, two-sided).

For the second set of experiments, the apparent diffusion coefficient of diffusion-weighted imaging sequences of the brain at 24 h after resuscitation was used. As the mean cortical apparent diffusion coefficient value was expected to be 0.47 ± 0.07 μm^2^/ms at 24 h after cardiac arrest^12^ and considering a 25% increase in the mean value in IDO^−/−^ mice, we anticipate that 6 mice per group were required for the magnetic resonance imaging study (α = 0.05, β = 0.8, two-sided).

All procedures involving animals were conducted in conformity with institutional guidelines, which adhere to the principles set out in the following laws, regulations, and policies governing the care and use of laboratory animals: Italian governing law (D.lgs 26/2014; authorization No. 19/2008-A issued March 6, 2008, by the Ministry of Health; Rome, Italy); Mario Negri institutional regulations and policies providing internal authorization for persons conducting animal experiments (Quality Management System Certificate – UNI EN ISO 9001:2015 – regulation No. 6121); the National Institutes of Health *Guide for the Care and Use of Laboratory Animals* (2011 edition); and European Union directives and guidelines (European Economic Community council directive No. 2010/63/UE). Approvals by the Istituto di Ricovero e Cura a Carattere Scientifico–Istituto di Ricerche Farmacologiche Mario Negri animal care and use committee and by the Italian Ministry of Health (D.lgs 738/2018-PR) were obtained.

### Animal Preparation

Two strains of mice were used for this study: 8- to 12-week-old, male C57BL/6 wild-type mice, and indoleamine 2,3-dioxygenase knock-out (IDO^−/−^) mice^13^ on a C57BL/6 background (body weight, 25 to 30 g). IDO^−/−^ mice were purchased from the Jackson Laboratories (USA), and then a colony was raised in our facility.

In response to peer review, additional behavioral data from female wild-type and IDO^−/−^ mice were obtained. The mice were intubated, mechanically ventilated (rodent ventilator 28025, Ugo Basile, Italy), and instrumented under anesthesia with isoflurane as previously described.^12^ The animals were ventilated with a respiratory rate of 110 to 120 breaths/min and a tidal volume of 10 μl/g of body weight. Anesthesia was maintained with isoflurane 1.5 to 2 vol% in 30% oxygen. Fluid-filled microcatheters (PE-10, Becton Dickinson, USA) were inserted into the left femoral artery and vein, respectively, for monitoring blood pressure and for drug administration. Invasive blood pressure and needle-probe electrocardiogram were recorded and analyzed with the use of a PC-based data acquisition system (Labchart 8.0, Powerlab ADInstruments, Dunedin, New Zealand).

### Mouse Model of Cardiac Arrest and CPR

Experimental cardiac arrest and CPR in mice were performed using an established model.^12,14^ Briefly, the mice were subjected to cardiac arrest induced by potassium chloride (0.08 mg/g IV). After 8 min of untreated cardiac arrest, finger chest compressions were delivered at a rate of 300/min. Mechanical ventilation with 100% oxygen was performed with a tidal volume of 10 μl/g at a rate of 100 breaths/min. Infusion of 0.6 µg min^−1^ epinephrine was initiated 30 s before starting CPR. Chest compressions were delivered until return of spontaneous circulation was achieved. Return of spontaneous circulation was defined as the return of sinus rhythm associated with mean arterial pressure (MAP) of greater than 40 mmHg lasting for at least 10 s. The mice were weaned from mechanical ventilation and extubated 60 min after CPR. Core body temperature was monitored by a rectal temperature probe and maintained at 37°C by a warming lamp before cardiac arrest and after return of spontaneous circulation. After incisional wound closure, the mice were returned to their cages (room air, ambient temperature of ~25°C, 12-h light and dark cycles) until the end of the study period. The survival times were recorded up to 7 days for the first set of experiments and for 24 h for the second set of experiments. For surgical pain relief during the recovery period, 2 mg/kg of 0.25% bupivacaine was infiltrated around the incisional wound after CPR. For pain relief, 0.1 mg/kg of buprenorphine intraperitoneally before and 12 h after the procedure were administrated.

### Assessment of Neurologic Function

The neurologic function score was assessed every 24 h for the 7 days of observation after resuscitation by an investigator blinded to the experimental groups. A neurologic function scoring system previously described was used with minor modifications.^14–16^ Five parameters were assessed and scored: level of consciousness (no reaction to pinching of tail = 0, poor response to tail pinch = 1, normal response to tail pinch = 2); corneal reflex (no blinking = 0, sluggish blinking = 1, normal blinking = 2); respirations (irregular breathing pattern = 0, decreased breathing frequency with normal pattern = 1, normal breathing frequency and pattern = 2); coordination (no movement = 0, moderate ataxia = 1, normal coordination = 2); and movement/activity (no spontaneous movement = 0, sluggish movement = 1, normal movement = 2). The total score was reported as the neurologic function score (total possible score = 10); dead mice were scored 0. Only surviving animals were included in the graph and in the statistical analysis (*i.e.*, n = 6 of 16 wild-type mice and n = 11 of 16 IDO^−/−^ mice at 7 days after cardiac arrest).

### Continuous Locomotor Activity

Spontaneous locomotor activity was measured continuously through a digital Ventilated cage system manufactured by Techniplast SpA (Italy). An activity metric, measured as the total distance walked, was recorded continuously in individually housed mice after cardiac arrest up to 7 days after resuscitation. The system consists of a standard individual ventilated cage and an electronic sensing board underneath each cage. The sensing board is composed by 12 electrodes connected to an integrated circuit that continuously measures their electrical capacitance. The electromagnetic field generated registered the effect of the presence of a mouse over an electrode that modifies the electromagnetic field, causing a drop of the electrode signal, due to the change in electrical capacitance. The electrical capacitance-sensing technology allows nonintrusive and continuous monitoring of the activity of the animals.^17^ The total distance traveled by each animal was computed for each day, and dark and light cycles were reported up to 7 days postresuscitation. The variation of locomotor activity from light to dark cycles was calculated as follows: (Activity_dark_ − Activity_light_)/Activity_light_ × 100. Activity metrics were reported for animals who survived up to 7 days after cardiac arrest with continuous data recording throughout the observation period (n = 4 of 6).

### Brain Magnetic Resonance Imaging Acquisitions and Analysis

To characterize post–cardiac arrest brain injury, diffusion-weighted imaging and diffusion tensor imaging sequences were acquired for quantification of cerebral edema and white matter damage respectively at 24 h and 7 days after cardiac arrest. The severity of white matter damage was quantified by comparing the values of fractional anisotropy and diffusivity (radial, axial) obtained from diffusion tensor imaging sequences. The apparent diffusion coefficient was used as a quantitative measurement of water diffusion changes in the brain. Intracerebral cytotoxic edema reduces water diffusivity, and this corresponded to a reduction in apparent diffusion coefficient values.

Brain imaging was performed on a 7 T small-bore animal scanner (Biospec; Bruker, Germany) running ParaVision 6.01 and equipped with a quadrature 1H CryoProb (Bruker) surface coil as transmitter and receiver. The anesthetized mice (isoflurane 1.5 to 2% in 30% oxygen) were positioned on the magnet. Respiratory frequency was monitored throughout the experiment, and the body temperature was maintained at 37°C with a water-heating pad.

Diffusion weighted echo-planar images (repetition time/echo time = 7,000/ 35 ms; image resolution of 100 × 100 μm^2^; field-of-view = 1.5 × 1.5 cm^2^; acquisition matrix = 150 × 150; slice thickness = 500 μm) were adopted to obtain the apparent diffusion coefficient maps. Diffusion encoding was applied in three orthogonal directions with b values of 0 and 1,300 s/mm^2^, respectively. Apparent diffusion coefficient maps were calculated on a pixel-by-pixel basis with an in-house MATLAB script using the model function: ln(S(b)/S0) = −b apparent diffusion coefficient, where S(b) is the measured signal intensity at a specific b value (b), and S0 is the signal intensity in the absence of a diffusion gradient (b = 0). An expert operator blind to experimental conditions extracted the apparent diffusion coefficient values in three regions of interest: frontal cortex, caudoputamen, and hippocampus using ITK-SNAP software (USA).^18^ Group average apparent diffusion coefficient values were reported for each region of interest, and the mean of the three analyzed regions was reported as the total. Six mice/group were required for this study based on an apparent diffusion coefficient value of 0.47 ± 0.07 μm^2^/ms in the cortex at 24 h after cardiac arrest^2^ and considering a 25% increase in the mean value in IDO^−/−^ mice (α = 0.05, β = 0.8, two-sided).

For diffusion tensor imaging, echo-planar imaging sequences were acquired (repetition time/echo time = 7,000/27.5 ms; image resolution of 125 × 125 μm^2^; field-of-view = 1.5 × 1.5 cm^2^; acquisition matrix = 120 × 120; slice thickness = 300 μm). Diffusion encoding b factors of 800 s/mm^2^ were applied along 19 isotropic directions with two b = 0 unweighted images for each repetition. The diffusion tensor was computed using FSL software (Oxford, United Kingdom). A group mean full tensor template was first created using a population-based diffusion tensor imaging atlas construction algorithm that adopts a tensor-based registration procedure embedded in the diffusion tensor imaging tool kit software library. Fractional anisotropy images of all subjects were normalized to the mean template with a diffeomorphic transformation, and the transformations were applied to all the diffusion tensor imaging diffusivity metrics (radial and axial), which were warped to the mean skeleton for region of interest-based analysis.

Quantitative magnetic resonance imaging variables used from diffusion tensor imaging sequences were axial and radial diffusivity and fractional anisotropy. Axial diffusivity represents the rate of water diffusion along the principal axis parallel to the main vector of the white matter fiber, while radial diffusivity indicates the rate of diffusion perpendicular to the main vector.^19^ These values reflect, respectively, axonal degeneration and myelin loss.^20,21^ Fractional anisotropy provides instead a cumulative direction of water diffusion, suggesting that a lower value of fractional anisotropy indicates a loss of directionality of water molecule diffusion in the white matter tracts, representing microstructural damage of the white matter fibers. Diffusion tensor imaging analysis was performed in wild-type mice (n = 4 of 6) and IDO^−/−^ animals (n = 5 of 6) at 7 days after cardiac arrest.

### Tissue Processing for Histological Analysis

The mice were deeply anaesthetized with isofluorane 5% in 30% oxygen and euthanized by guillotine. The brains were harvested and carefully removed from the skull, fixed in 10% buffered formalin, and paraffin-embedded for immunohistochemistry.

Immunohistochemistry was done on 8-μm brain sagittal sections incubated overnight at 4°C with anti-IBA1 (1:200; Wako, Neuss, Germany) to detect microglia macrophage activation and with primary monoclonal antibody mouse anti-mouse glial fibrillary acid protein (1:2,000; Millipore, USA). The sections were then exposed to anti-rabbit biotinylated secondary antibody (1:200; Vector Laboratories Inc., USA). Positive cells were stained by reaction with 3,3-diaminobenzidine tetrahydrochloride (Vector Laboratories Inc.). Negative control studies, without the primary antibody, were performed in parallel.

The entire brain sections were acquired at 20× by an Olympus BX-61 virtual stage microscope, with a pixel size of 0.346 μm. Acquisition was done greater than 5-μm-thick stacks, with a step size of 1 μm. The different focal planes were merged into a single stack by mean intensity projection to ensure consistent focus throughout the sample. For immunostaining quantifications, the regions of interest were corpus callosum, cortex, hippocampus, and caudoputamen.

The images were analyzed using Fiji software. For IBA1 and glial fibrillary acidic protein, both yielding a sharp signal-to-noise ratio, the positive signal was segmented by applying a gray-level threshold cutting-off the background. The immunostained areas were expressed as positive or total assessed pixels and reported as the percentages of the total stained area as previously described.^22^ A size threshold of more than 25 mm^2^ was used to select cells to be analyzed for area and perimeter. Mean single-cell values for each parameter were used for statistics. Fluoro-Jade labeling was used to detect degenerating neurons as previously described (6). Degenerating neurons were quantified by reckoning the number of the Fluoro-Jade–positive neurons in the regions of interest. The data obtained in each slice per brain area were averaged, thus providing a single value for each brain area, and this value was used for statistical analysis. Histologic examination was performed in surviving animals from both the first and the second sets of experiments. In mice surviving up to 7 days after cardiac arrest, microglia and macrophage activation and astrogliosis was assessed in wild-type mice (n = 4 to 6 of 6) and IDO^−/−^ animals (n = 2 of 6 of 11). In mice surviving 24 h after cardiac arrest, degenerating neurons by Fluoro-Jade staining were quantified in wild-type (n = 3 to 4 of 6), IDO^−/−^ mice (n = 4 to 6 of 6), and IDO^−/−^ animals injected with l-kynurenine (n = 2 to 4 of 6).

### Administration of l-Kynurenine

l-Kynurenine (Sigma–Aldrich, Italy) was administered as described previously.^23^ Briefly, l-kynurenine was dissolved in 0.1 M HCl and brought to a final concentration of 2 mg/mL in phosphate-buffered saline after adjusting pH to 6.5 to 7.0. The mice were injected with l-kynurenine (20 mg/kg iv) 15 min before cardiac arrest induction. The compared groups, wild-type and IDO^−/−^ mice, received the same volume of phosphate-buffered saline intravenously 15 min before cardiac arrest. Cardiac arrest was induced in 44 IDO^−/−^ mice injected with l-kynurenine, 15 wild-type mice, and 10 IDO^−/−^ animals.

### Kynurenine Pathway Metabolites and Biomarker Measurements

Quantification of plasmatic tryptophan, kynurenine, and kynurenic acid was performed following the high-performance liquid chromatography (HPLC)–tandem mass spectrometry methods previously described for simultaneously detection of these analytes.^7^ To measure plasma kynurenine pathway metabolites levels after cardiac arrest, terminal blood withdrawal was performed. Venous blood was obtained *via* inferior vena cava puncture in anesthetized mice using a heparinized syringe 1 day and 7 days after return of spontaneous circulation in wild-type and IDO^−/−^ mice. Control blood samples were obtained from naïve mice that were not subjected to cardiac arrest.

Blood samples were centrifuged at 2,000*g* for 15 min at 4°C, and plasma was stored at −80°C until analysis. Methanol (MeOH), acetic acid (CH_3_COOH), acetonitrile (ACN), and formic acid (HCOOH) were from Sigma–Aldrich; all solvents were of liquid chromatography–mass spectrometry grade. Liquid chromatography–mass spectrometry grade water was obtained in-house with a Milli-Q system (Millipore). l-Tryptophan, l-kynurenine, and kynurenic acid were from Sigma–Aldrich. KYN-d4 was from Buchem BV (The Netherlands), and TRP-d5 and KYNA-d5 were from CDN Isotopes (Italy).

Quantification of plasmatic tryptophan, kynurenine, and kynurenic acid was performed following the HPLC–tandem mass spectrometry methods previously described for simultaneously detection of these analytes.^7^ Briefly, plasma samples were fortified with deuterated internal standards and then mixed with cold methanol and incubated at −20°C for protein precipitation before centrifugation at 13,000*g* for 10 min (at 4°C). The supernatant was then dried under N_2_, and the residue was resuspended and injected into the HPLC system (Alliance 2695, Waters Corp., United Kingdom). Separation was done after a gradient elution on an Accucore pentafluorophenyl column (Thermo Scientific, USA), and mass spectrometric analysis was done with a Quattro Micro API triple quadrupole instrument (Waters Corp.) in positive ion mode and multiple reaction monitoring mode, measuring the fragmentation products of the deprotonated pseudomolecular ions. The HPLC–tandem mass spectrometry system was controlled by MassLynx version 4.1 (Waters Corp.), and the data were collected with the same software. In each analytical run, a six-point calibration curve linear in the ranges 0.625 to 25 µg/mL, 0.025 to 1 µg/mL, and 1.2 to 48 ng/mL for tryptophan, kynurenine, and kynurenic acid, respectively, run in parallel with the unknown and quality control samples.

A novel axonal biomarker, neurofilament light chain, was measured in plasma with an ultrasensitive single molecule array after cardiac arrest. Terminal blood withdrawal was performed to measure plasma neurofilament light chain levels after cardiac arrest. Venous blood was obtained *via* inferior vena cava puncture in anesthetized mice using a heparinized syringe at 7 days after return of spontaneous circulation in wild-type and IDO^−/−^ mice. Control blood samples were obtained from naïve mice that were not subjected to cardiac arrest (n = 7). Neurofilament light chain levels were measured using the single-molecule array Quanterix SIMOA NF-light kit and SIMOA HD-1 analyzer (Quanterix Corporation, USA).^24^ The plasma neurofilament light chain concentrations were expressed in picograms per millilitre (pg/mL). Kynurenine pathway metabolites and neurofilament light chain concentrations were quantified at 7 days after cardiac arrest in surviving wild-type mice (n = 3 to 5 of 6) and IDO^−/−^ animals (n = 4 to 7 of 11).

### Statistical Analysis

The animals were randomized on 1:1 or 1:1:1 basis with a list randomizer (https://www.random.org/lists/) according to the treatment group in two independent experiments. For the primary aim, we wished to test the effect of genetic deletion of indoleamine 2,3-dioxygenase on survival up to 7 days after cardiac arrest. For the secondary aim, we wished to determine the ability of the genetic deletion of the enzyme to prevent brain injury and to preserve neurologic function up to 7 days after cardiac arrest. For the primary and secondary aim, the following groups were compared: treatment group, indoleamine 2,3-dioxygenase knockout mice (IDO^−/−^) *versus* control group, wild-type mice (wild-type).

Furthermore, we wished to assess whether l-kynurenine could be responsible for the neurotoxic effect of indoleamine 2,3-dioxygenase activation early after resuscitation. For the second sets of experiments on phenotype rescuing, the following groups were compared: treatment group, indoleamine 2,3-dioxygenase knockout mice (IDO^−/−^) *versus* control group, wild-type mice *versus* a positive control group, IDO^−/−^ + l-kynurenine.

Kaplan–Meier analysis and log-rank test were used to calculate the survival rates (primary outcome). Differences in survival were further explored by using the Cox proportional hazard modeling. The normality of data distribution was assessed using the D’Agostino–Pearson omnibus normality test. Continuous and categorical data variables were described as mean ± SD of mean or median (interquartiles) as appropriate.

Differences among groups for continuous data were assessed using unpaired Student’s *t* test or Mann–Whitney U test, according to data distribution. Differences in categorical data among groups were assessed using Fisher’s exact test or chi-square test. Differences between the three study groups were evaluated with using a one-way ANOVA or Kruskall–Wallis according to data distribution. Only in the presence of a significant one-way ANOVA, were *post hoc* multiple comparisons between groups at different time points performed by controlling the false discovery rate using the two-stage step-up method of Benjamini, Krieger, and Yekutieli. Differences between the study groups over time were evaluated using mixed-effects models for repeated measurement analysis or by using ordinary two-way ANOVA for independent measures. Mixed-effects models were performed by considering time, treatment (*i.e.*, mice group), and time by treatment interaction as fixed effects. Subjects (*i.e.*, mice) were considered as a random effect.

The treatment effect of mixed effects models or ordinary two-way ANOVA was reported. *Post hoc* multiple comparisons between groups at different time points were performed by controlling the false discovery rate using the two-stage step-up method of Benjamini, Krieger, and Yekutieli in the presence of a treatment effect *P* < 0.05 (two-tailed). Significance was considered at the level of *P* < 0.05 (two-tailed). GraphPad Prism 8.0 (GraphPad Software Inc., USA), and STATA-14/MP (StataCorp LP, USA) was used for statistical analyses.

## Results

### Genetic Deletion of Indoleamine 2,3-Dioxygenase Improves Survival and Prevents Post−cardiac arrest Brain Injury

#### Survival and Neurologic and Functional Recovery

**Fig. 1.**
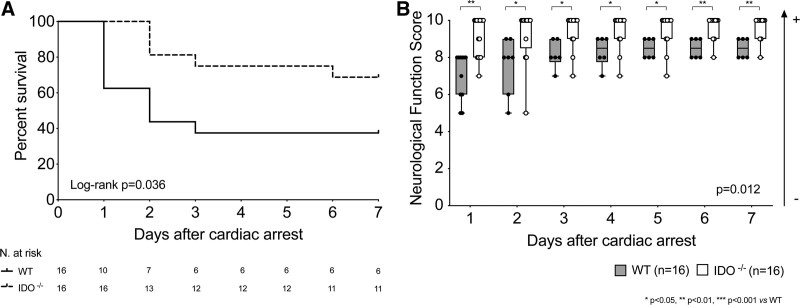
(*A*) Survival rate and (*B*) neurologic function score in surviving mice during the first 7 days after cardiac arrest and cardiopulmonary resuscitation (*P* = 0.036 *versus* wild type (WT) by log-rank test; n = 16 animals/group). Treatment effect *P* values of the mixed effects models for repeated measurement analysis are reported. *Post hoc* multiple comparisons were performed by controlling the false discovery rate using the two-stage step-up method of Benjamini, Krieger, and Yekutieli (n = 16 animals/group). *, *P* < 0.05; **, *P* < 0.01 *versus* wild-type group. IDO^−/−^ indicates knock-out mice for indoleamine 2,3-deoxygenase.

The survival rate was 37.5% (6 of 16) in wild-type mice at 7 days after resuscitation. IDO^−/−^ animals showed significantly higher survival of 68.75% (11 of 16; log-rank *P* = 0.036, n = 16/group; fig. 1A). The hazard ratio for death was 0.36 (95% CI, 0.12 to 1.06; *P* = 0.064, n = 32). The neurologic function score was significantly better in IDO^−/−^ mice than in the wild-type animals throughout the 7 days after cardiac arrest (mean neurologic function score: wild-type 7 ± 1 *versus* IDO^−/−^ 9 ± 1, *P* = 0.012, n = 16/group; fig. 1B).

**Fig. 2.**
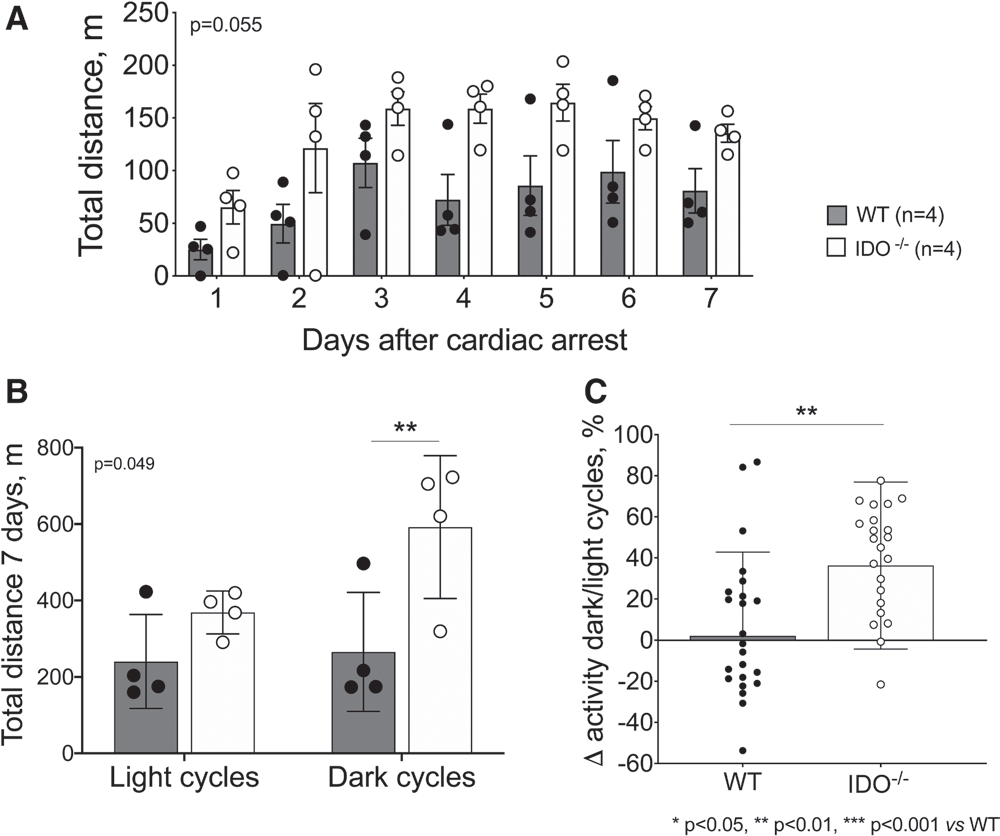
Spontaneous locomotor activity in the 7 days after cardiac arrest or cardiopulmonary resuscitation. (*A*) Total distance traveled daily in wild-type (WT) and IDO^−/−^ mice. Treatment effect *P* values of the mixed effects models for repeated measurement analysis are reported (n = 4 animals/group). (*B*) Total distance covered during the light and dark phases by wild-type and IDO^−/−^ mice. Treatment effect *P* values of the mixed effects models for repeated measurement analysis are reported. *Post hoc* multiple comparisons were performed by controlling the false discovery rate using the two-stage step-up method of Benjamini, Krieger, and Yekutieli. **, *P* < 0.01 *versus* wild-type group. (*C*) Variation of locomotor activity during the dark and light cycles, expressed as a percentage: (Activity_dark_ − Activity_light_)/Activity_light_ × 100 (n = 24 observations/group). **, *P* < 0.01 *versus* wild-type group by Mann–Whitney U test. IDO^−/−^ indicates knock-out mice for indoleamine 2,3-deoxygenase.

Spontaneous locomotor activity continuously recorded in cardiac arrest mice is reported in figure 2 and supplemental figure 1 (https://links.lww.com/ALN/D241). After cardiac arrest, wild-type mice exhibited a decline in activity compared to indoleamine 2,3-dioxygenase–deleted animals during the 7 days after resuscitation (total distance traveled in 7 days: wild-type 506 ± 278 m *versus* IDO^−/−^ 963 ± 242 m; *P* = 0.055, n = 4/group; fig. 2A). In IDO^−/−^ mice, the physiologic cyclical variation of locomotor activity was observed every 12 h, scoring lower during the light phases and higher during the dark phases. These cyclic differences disappeared in wild-type animals with cardiac arrest. While the total distance traveled by the animals did not differ during the light phases between the two groups, during the dark phases, it was significantly lower in wild-type mice compared with IDO^−/−^ animals (total distance traveled in dark phases: wild-type 265 ± 155 m *versus* IDO^−/−^ 592 ± 187 m; n = 4/group, *P* = 0.006; fig. 2B). As shown, locomotor activity increased by 36% during the dark phases in IDO^−/−^ mice, while it declined up to 2% in wild-type animals (change in activity during dark and light cycles: wild-type 2 ± 41% *versus* IDO^−/−^ 36 ± 41%; *P* = 0.001; fig. 2C). Data about neurologic function score and locomotor activity were reported also in wild-type and IDO^−/−^ mice without cardiac arrest, showing no differences among the two groups (supplemental fig. 2, https://links.lww.com/ALN/D242; supplemental fig. 3, https://links.lww.com/ALN/D243).

#### CPR and Hemodynamics

No significant differences in body weight, temperature, total dose of epinephrine and CPR time to resuscitation were observed between wild-type and IDO^−/−^ mice. At baseline, hemodynamic parameters including heart rate and MAP did not differ among groups (table 1; supplemental table, https://links.lww.com/ALN/D252). Heart rate did not differ significantly over time between the two groups, while MAP at the time of resuscitation and at 30 and 60 min after resuscitation was significantly higher in IDO^−/−^ mice compared with wild-type mice (table 1).

**Table 1.**
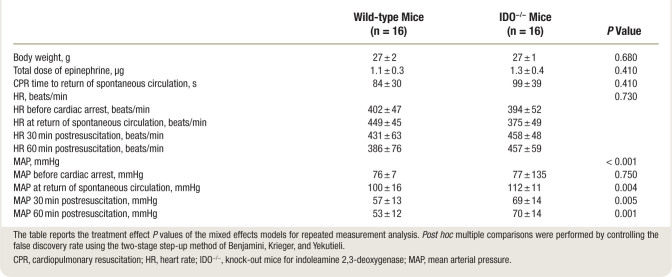
CPR Characteristics and Hemodynamic Parameters

#### Kynurenine Pathway Metabolites and Biomarker Measurements

**Fig. 3.**
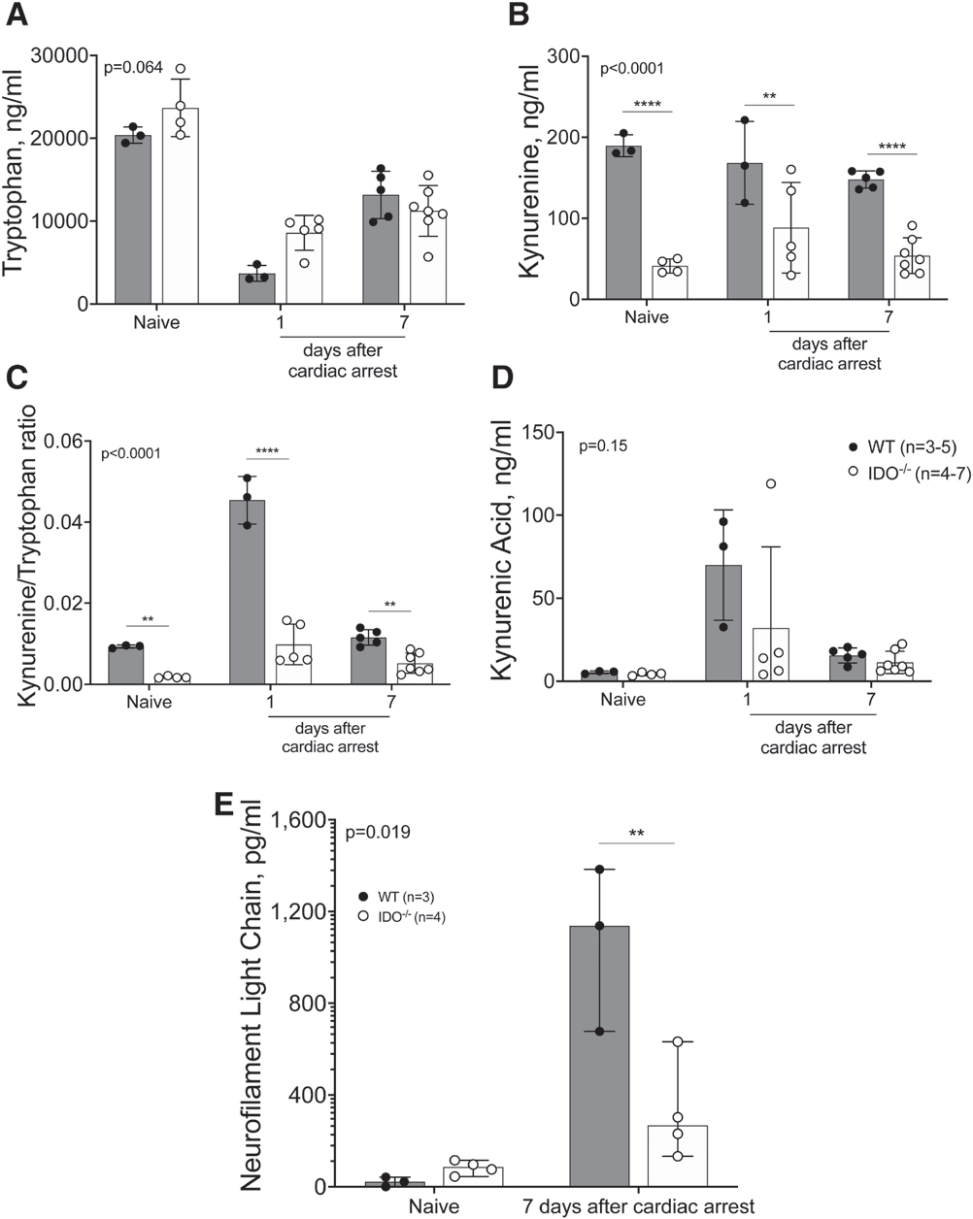
Kynurenine pathway metabolites concentration in naïve mice (n = 3 to 4 for each group); and at days 1 and 7 after cardiac arrest (n = 3 to 7 for each group). (*A* to *D*) Plasmatic levels of neurofilament light chain in naïve mice and at 7 days after cardiac arrest. (*E*) Treatment effect *P* values of the mixed effects models for repeated measurement analysis are reported. *Post hoc* multiple comparisons were performed by controlling the false discovery rate using the two-stage step-up method of Benjamini, Krieger, and Yekutieli. *, *P* < 0.05; **, *P* < 0.01; ***, *P* < 0.001; ****, *P* < 0.0001 *versus* wild-type (WT) group. IDO^−/−^ indicates knock-out mice for indoleamine 2,3-deoxygenase.

We explored the kynurenine pathway by measuring metabolites in naïve mice and in wild-type and IDO^−/−^ animals at 1 and 7 days after cardiac arrest (fig. 3, A to D). At baseline, when compared to wild-type mice, IDO^−/−^ mice show equivalent tryptophan concentrations and profound depletion of kynurenine ([tryptophan] in naïve wild-type *versus* IDO^−/−^ mice, 20,380 ± 985 ng/mL *versus* 23,662 ± 3,477 ng/mL; *P* = 0.064; [kynurenine] in naïve wild-type *versus* IDO^−/−^ mice, 190 ± 13 ng/mL *versus* 41 ± 9 ng/mL; *P* < 0.001; n = 3 to 5/group; fig. 3, A and B). After resuscitation, a significant increase in kynurenine/tryptophan ratio was observed in wild-type mice compared to IDO^−/−^ mice at 1 day after cardiac arrest ([kynurenine/tryptophan ratio] in wild-type *versus* IDO^−/−^ mice, 0.05 ± 0.01 *versus* 0.01 ± 0.01; *P* < 0.001; n = 3 to 5/group; fig. 3C). Furthermore, a higher kynurenine/tryptophan ratio is associated with neurologic impairment at 1 and 7 days after cardiac arrest (supplemental fig. 2, https://links.lww.com/ALN/D242).

The neurofilament light concentrations were significantly higher in wild-type mice compared to IDO^−/−^ mice after resuscitation. At 7 days after cardiac arrest, the median concentrations (interquartile range) were 1,138 pg/mL (678 to 1,384) in wild-type mice compared to 267 (157 to 550) pg/mL for IDO^−/−^ mice (*P* < 0.001; n = 3 to 4/group; fig. 3E).

#### Cerebral White Matter Injury on Magnetic Resonance Imaging

**Fig. 4.**
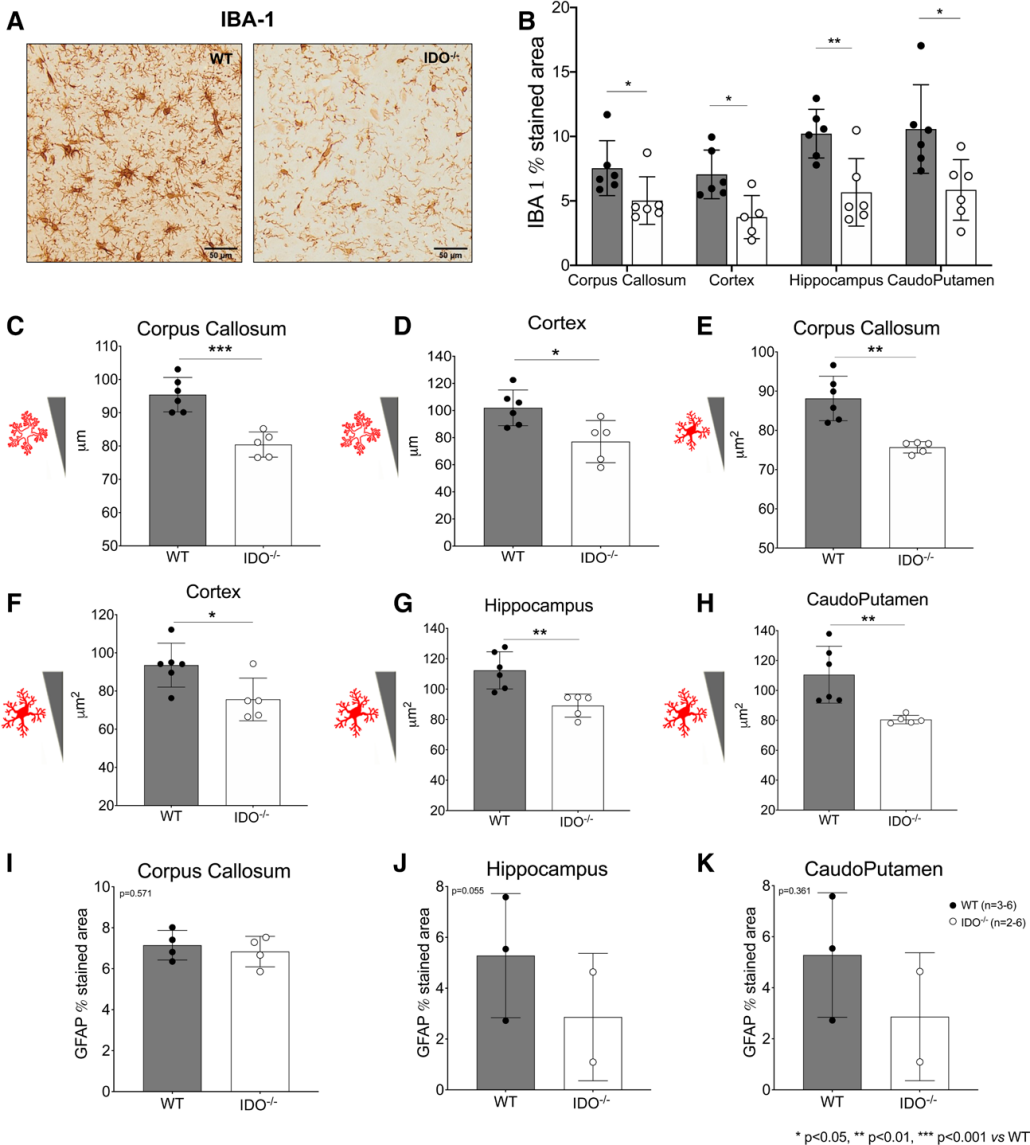
Diffusion tensor magnetic resonance imaging of mice 7 days after cardiac arrest with diffusivity analysis. (*A*) Representative three-dimensional reconstruction of white matter tracts (corpus callosum and internal and external capsule). (*B* to *D*) Average values of fractional anisotropy in corpus callosum, external capsule, and internal capsule. (*E* to *G*) Average values of radial diffusivity in corpus callosum, external capsule, and internal capsule. (*H* to *J*) Average values of axial diffusivity in corpus callosum, external capsule, and internal capsule. IDO^−/−^ indicates knock-out mice for indoleamine 2,3-deoxygenase. *, *P* < 0.05; **, *P* < 0.01 *versus* wild-type group by unpaired *t* test or Mann–Whitney U test, n = 4 wild-type mice, n = 5 IDO^−/−^ mice.

Diffusion tensor magnetic resonance imaging sequences were obtained from wild-type and IDO^−/−^ animals at 7 days after cardiac arrest. Figure 4A shows the three-dimensional reconstruction of white matter tracts (corpus callosum and internal and external capsule) obtained from diffusion tensor imaging sequences and used for quantitative analysis. Overall, indoleamine 2,3-dioxygenase deletion exerted a protective effect on white matter disruption at 7 days after resuscitation.

IDO^−/−^ mice showed significantly higher values of fractional anisotropy compared to wild-type animals in the corpus callosum (IDO^−/−^ 0.68 ± 0.01 *versus* wild-type 0.65 ± 0.01, *P* = 0.010, n = 4 to 5/group) and in the external capsule (IDO^−/−^ 0.47 ± 0.01 *versus* wild-type 0.45 ± 0.01, *P* = 0.006, n = 4 to 5/group). In the internal capsule, a tendency toward an increase in fractional anisotropy was observed in the IDO^−/−^ group compared to the wild-type one (IDO^−/−^ 0.65 ± 0.02 *versus* wild-type 0.61 ± 0.03, *P* = 0.070, n = 4 to 5/group). The mean radial diffusivity value was 0.46 ± 0.01 μm^2^/ms for the IDO^−/−^ group and 0.48 ± 0.01 μm^2^/ms for the wild-type one in the external capsule (*P* = 0.065). An increase in axial diffusivity in the corpus callosum was observed in IDO^−/−^ mice compared to wild-type animals (mean axial diffusivity: IDO^−/−^ 1.24 ± 0.03 μm^2^/ms *versus* wild-type 1.20 ± 0.01 μm^2^/ms; *P* = 0.029, n = 4 to 5/group; fig. 4H).

#### Microglia and Macrophage Activation and Astrogliosis

**Fig. 5.**
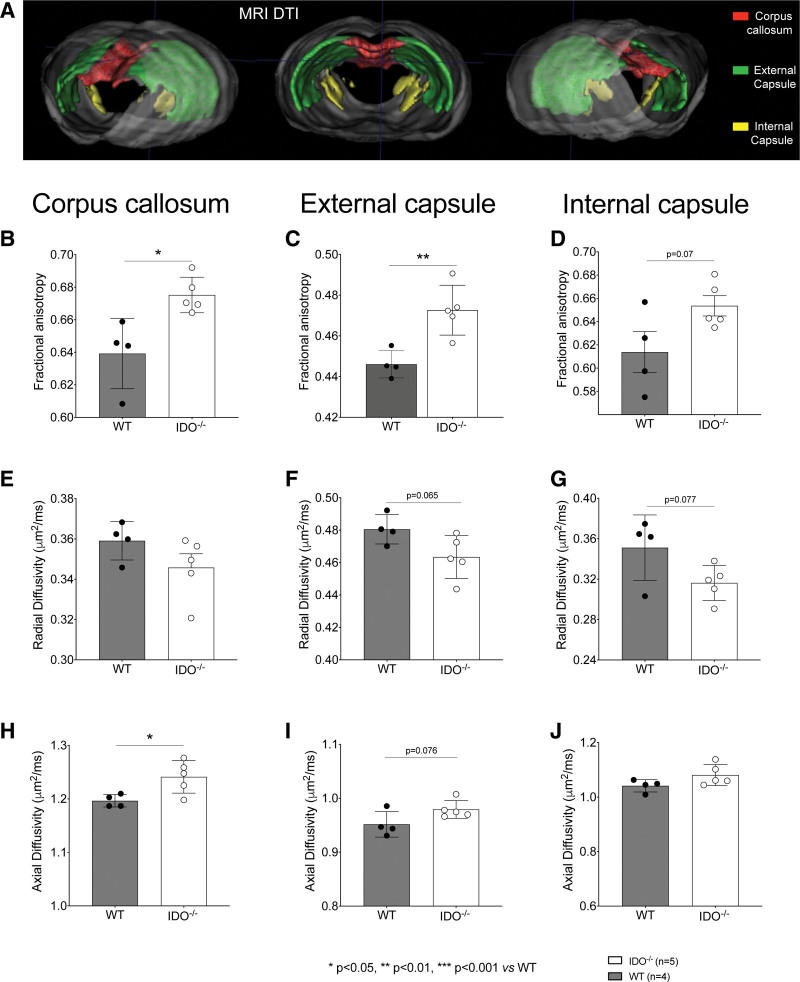
(*A*) Representative images of IBA1 immunostaining. (*B*) Quantification of IBA1-stained areas in different brain areas (corpus callosum, cortex, hippocampus, and caudoputamen) of mice 7 days after cardiac arrest (n = 6 wild-type (WT) mice, n = 5 to 6 IDO^−/−^ mice). (*C* to *H*) Quantification of shape parameters for IBA1-positive cells including perimeter in the corpus callosum (n = 6 wild-type mice, n = 5 IDO^−/−^ mice; *C*) and cortex (n = 6 wild-type mice, n = 5 IDO^−/−^ mice; *D*) and area in the corpus callosum (n = 6 wild-type mice, n = 5 IDO^−/−^ mice; *E*), cortex (n = 6 wild-type mice, n = 5 IDO^−/−^ mice; *F*), hippocampus (n = 6 wild-type mice, n = 5 IDO^−/−^ mice; *G*), and caudoputamen (n = 6 wild-type mice, n = 5 IDO^−/−^ mice; *H*). (*I* to *K*) Quantification of glial fibrillary acid protein–stained area in corpus callosum (n = 4 wild-type mice, n = 4 IDO^−/−^ mice; *I*), hippocampus (n = 3 wild-type mice, n = 2 IDO^−/−^ mice; *J*), and caudoputamen (n = 3 wild-type mice, n = 2 IDO^−/−^ mice; *K*). IDO^−/−^ indicates knock-out mice for indoleamine 2,3-deoxygenase. *, *P* < 0.05; **, *P* < 0.01; ***, *P* < 0.001 *versus* wild-type group by unpaired *t* test or Mann–Whitney U test.

Seven days after cardiac arrest, a marked reduction of the areas stained with ionized calcium-binding adaptor molecule 1 (IBA1) was observed in IDO^−/−^ animals compared to wild-type mice in different areas of the brain, as shown in figure 5 (A and B). Specifically, the IBA1-stained area was significantly reduced in IDO^−/−^ mice compared to wild-type group in the corpus callosum (IDO^−/−^ 5 ± 2% *versus* wild-type 8 ± 2%; *P* = 0.040, n = 6/group), in the frontal cortex (IDO^−/−^ 4 ± 2% *versus* wild-type 7 ± 2%; *P* = 0.010, n = 5 to 6/group), in the hippocampus (IDO^−/−^ 6 ± 3% *versus* wild-type 10 ± 2%; *P* = 0.006, n = 6/group), and in the caudoputamen (IDO^−/−^ 6 ± 2% *versus* wild-type 11 ± 3%; *P* = 0.020, n = 6/group).

Shape parameters for IBA1-positive cells indicated a prevalence of ramified cells 7 days after cardiac arrest in wild-type mice, while indoleamine 2,3-dioxygenase deletion was accompanied by a less toxic phenotype of microglial cells (fig. 5, C to H). Quantification of these parameters showed that the mean perimeter of IBA1-positive cells was significantly lower in IDO^−/−^ mice compared to wild-type animals in the corpus callosum (IDO^−/−^ 80 ± 4 μm *versus* wild-type 95 ± 5 μm, *P* < 0.001, n = 5 to 6/group; fig. 5C) and in the frontal cortex (IDO^−/−^ 77 ± 16 μm *versus* wild-type 102 ± 13 μm; *P* = 0.018, n = 5 to 6/group; fig. 5D). The mean area of IBA1-positive cells was significantly lower in IDO^−/−^ animals compared to wild-type mice in the corpus callosum (IDO^−/−^ 76 ± 2 μm^2^ *versus* wild-type 88 ± 6 μm^2^; *P* = 0.001, n = 5 to 6/group; fig. 5E), in the frontal cortex (IDO^−/−^ 76 ± 11 μm^2^ *versus* wild-type 94 ± 12 μm^2^; *P* = 0.020, n = 5 to 6/group; fig. 5F), in the hippocampus (IDO^−/−^ 89 ± 8 μm^2^ *versus* wild-type 112 ± 12 μm^2^; *P* = 0.005, n = 5 to 6/group; fig. 5G), and in the caudoputamen (IDO^−/−^ 81 ± 3 μm^2^ *versus* wild-type 111 ± 19 μm^2^; *P* = 0.007, n = 5 to 6/group; fig. 5H).

Astrogliosis marked by glial fibrillary acidic protein area staining at 7 days is represented in figure 5 (I to K). In IDO^−/−^ mice, a trend toward a reduction in the glial fibrillary acidic protein area staining was observed in the hippocampus at 7 days after resuscitation compared to wild-type animals (IDO^−/−^ 3 ± 2% *versus* wild-type 5 ± 2%; *P* = 0.055, n = 2 to 3/group; fig. 5J).

### l-Kynurenine Worsens Survival and Brain Edema after Cardiac Arrest

#### Survival and Neurologic Function

In this set of experiments, we tested the hypothesis that indoleamine 2,3-dioxygenase exerted its deleterious effect on brain function through kynurenine by injecting IDO^−/−^ mice with l-kynurenine before cardiac arrest induction. As shown in supplemental figure 7A (https://links.lww.com/ALN/D247), kynurenine levels were significantly increased in plasma after cardiac arrest in IDO^−/−^ mice injected with l-kynurenine compared with naïve IDO^−/−^ mice ([kynurenine] in naïve IDO^−/−^ *versus* IDO^−/−^+ l-kynurenine mice 30 min postresuscitation: 41 ± 9 ng/mL *versus* 5,716 ± 4,157 ng/mL; *P* = 0.011; n = 4 to 8/group). After resuscitation, a significant increase in kynurenine/tryptophan ratio was also observed in IDO^−/−^ + l-kynurenine mice compared to IDO^−/−^ mice ([kynurenine/tryptophan ratio] in naïve IDO^−/−^ *versus* IDO^−/−^+ l-kynurenine mice 30 min postresuscitation: 0.002 ± 0.001 *versus* 0.92 ± 0.54; *P* = 0.041; n = 4 to 8/group; supplemental fig. 7B, https://links.lww.com/ALN/D247); while the levels of the downstream neuroprotective metabolite kynurenic acid did not change significantly after cardiac arrest ([kynurenic acid] in naïve IDO^−/−^ *versus* IDO^−/−^+ l-kynurenine mice 30 min postresuscitation: 4.37 ± 1 ng/mL *versus* 2,147 ± 2,928 ng/mL; *P* = 0.247, n = 4 to 8/group; supplemental fig. 7C, https://links.lww.com/ALN/D247).

**Fig. 6.**
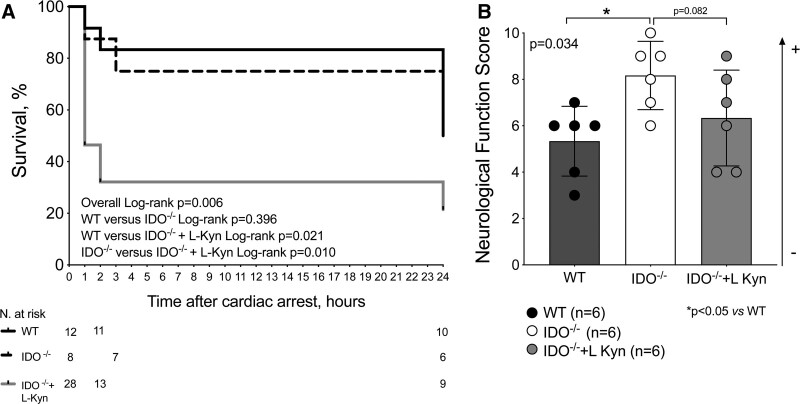
Survival rate and neurologic function score in surviving mice at 24 h after cardiac arrest and cardiopulmonary resuscitation. (*A*) Survival rate up to 24 h after cardiac arrest (*P* = 0.006 overall log-rank test, n = 12 wild-type (WT) mice, n = 8 IDO^−/−^ mice, n = 28 IDO^−/−^ + l-kynurenine animals). (*B*) Neurologic function score 24 h after cardiac arrest. The differences between the three study groups were evaluated with using a one-way ANOVA. *Post hoc* multiple comparisons were performed by controlling the false discovery rate using the two-stage step-up method of Benjamini, Krieger, and Yekutieli (n = 6 animals/group). *, *P* < 0.05 *versus* wild-type group. IDO^−/−^ indicates knock-out mice for indoleamine 2,3-deoxygenase.

The rate of spontaneous circulation was 80% in the two groups of mice wild-type (12 of 15) and IDO^−/−^ (8 of 10), and 64% (28 of 44) in IDO^−/−^ injected with l-kynurenine (*P* = 0.365; supplemental fig. 8, https://links.lww.com/ALN/D248). The survival rate at 24 h after resuscitation was 50% (6 of 12) in wild-type mice and 75% in IDO^−/−^ animals (6 of 8; fig. 6A). IDO^−/−^ mice injected with l-kynurenine showed significantly lower survival of 21% at 24 h after cardiac arrest (6 of 28, overall log-rank *P* = 0.006; fig. 6A). The overall hazard ratio for death was 0.45 (95% CI, 0.24 to 0.84; *P* = 0.012, n = 48). The neurologic function score was significantly better in IDO^−/−^ mice than in the wild-type animals at 24 h after cardiac arrest (neurologic function score: IDO^−/−^ 8 ± 1 *versus* wild-type 5 ± 2; *P* = 0.024, n = 6/group; fig. 6B). A trend toward a reduction in neurologic function score was observed in the group IDO^−/−^ + l-kynurenine compared to IDO^−/−^ animals (neurologic function score: IDO^−/−^ + l-kynurenine 6 ± 2 *versus* IDO^−/−^ 8 ± 1; *P* = 0.082, n = 6/group; fig. 6B).

Quantitative analysis of Fluoro-Jade–stained neurons revealed higher levels of degenerating neurons in the CA1 pyramidal cell layer of the hippocampus in wild-type and IDO^−/−^ + l-kynurenine compared to IDO^−/−^ animals at 24 h after resuscitation (supplemental fig. 9C, https://links.lww.com/ALN/D249). Fluoro-Jade reactivity was undetectable in sham-operated mice (data not shown).

#### Brain Water Diffusion

**Fig. 7.**
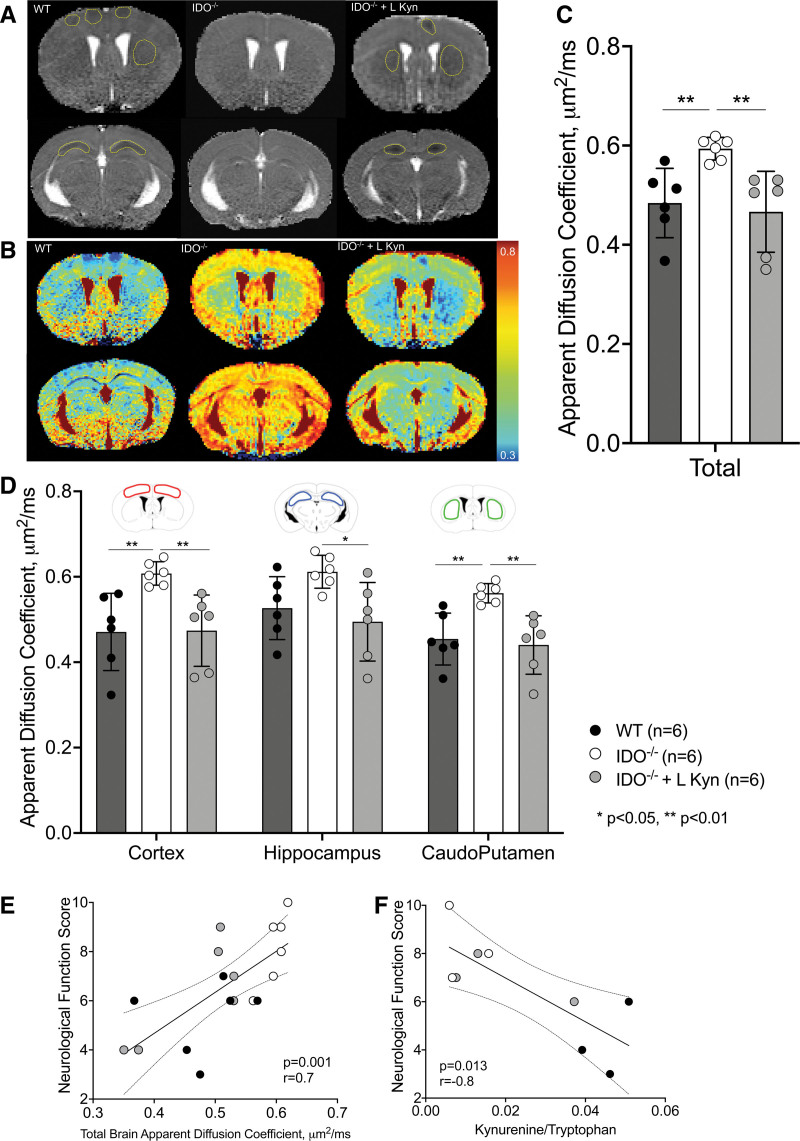
(*A*, *B*) Representative diffusion-weighted imaging maps in gray (*A*) and colorimetric scale (*B*) of mice 24 h after cardiac arrest. The *color bar* on the right side represents the color code for apparent diffusion coefficient values (μm^2^/ms). (*C*) Total brain apparent diffusion coefficient values of wild-type (WT) mice, IDO^−/−^ mice, and IDO^−/−^ + l-kynurenine mice are reported (n = 6 animals/group). (*D*) Apparent diffusion coefficient of each region of interest: cortex, hippocampus, and caudoputamenin (n = 6 animals/group). (*E*) Correlation between total brain apparent diffusion coefficient and neurologic function score at 24 h after cardiac arrest (n = 6 animals/group). (*F*) Correlation between levels of kynurenine/tryptophan ratio and neurologic function score at 24 h after cardiac arrest in the three groups: wild-type mice (n = 3), IDO^−/−^ mice (n = 3), and IDO^−/−^ + l-kynurenine mice (n = 3). The differences between the three study groups were evaluated with using a one-way ANOVA. Only in the presence of a significant one-way ANOVA were *post hoc* multiple comparisons between groups performed by controlling the false discovery rate using the two-stage step-up method of Benjamini, Krieger, and Yekutieli. *, *P* < 0.05; **, *P* < 0.01. IDO^−/−^ indicates knock-out mice for indoleamine 2,3-deoxygenase.

Figure 7 shows magnetic resonance images acquired 24 h after resuscitation, with apparent diffusion coefficient maps in gray and colorimetric scale. At 24 h after cardiac arrest, wild-type mice showed a restriction in water diffusion in each region of interest and across the whole brain (fig. 7C; supplemental Figure 5, https://links.lww.com/ALN/D245). IDO^−/−^ mice, instead, showed a protection against the development of ischemia-induced brain edema (fig. 7C). Specifically, apparent diffusion coefficient values were higher in the frontal cortex and caudoputamen in the IDO^−/−^ group compared to the wild-type group (fig. 7D). This beneficial effect was reverted by the administration of l-kynurenine before the induction of cardiac arrest in the frontal cortex, hippocampus, and caudoputamen and across the whole brain (apparent diffusion coefficient frontal cortex, μm^2^/ms: wild-type 0.47 ± 0.09, IDO^−/−^ 0.61 ± 0.03, IDO^−/−^ + l-kynurenine 0.47 ± 0.08; apparent diffusion coefficient hippocampus, μm^2^/ms: wild-type 0.53 ± 0.07, IDO^−/−^ 0.61 ± 0.04, IDO^−/−^ + l-kynurenine 0.49 ± 0.09; apparent diffusion coefficient caudoputamen, μm^2^/ms: wild-type 0.45 ± 0.06, IDO^−/−^ 0.56 ± 0.02, IDO^−/−^ + l-kynurenine 0.44 ± 0.07; apparent diffusion coefficient total brain, μm^2^/ms: wild-type 0.48 ± 0.07, IDO^−/−^ 0.59 ± 0.02, IDO^−/−^ + l-kynurenine 0.47 ± 0.08; fig. 7D; n = 6/group). At 24 h after cardiac arrest, a strong positive correlation was observed between the apparent diffusion coefficient of the total brain and the neurologic function score (r = 0.71, *P* = 0.001; fig. 7E). A strong negative correlation was observed between the kynurenine/tryptophan ratio and the neurologic function score (r = −0.80, *P* = 0.013; fig. 7F).

## Discussion

This study provides evidence that the kynurenine pathway is involved in the pathophysiology of post–cardiac arrest brain injury. Indeed, interfering with kynurenine pathway through indoleamine 2,3-dioxygenase deletion markedly improved survival and neurologic outcome after cardiac arrest. Indoleamine 2,3-dioxygenase deletion accelerated the recovery of spontaneous locomotor function while maintaining the physiologic circadian rhythm of the IDO^−/−^ animals after cardiac arrest. The neuroprotective effects of indoleamine 2,3-dioxygenase deletion were associated with preservation of white matter microintegrity and reduced axonal degeneration and demyelination, as shown by brain magnetic resonance imaging with diffusion tensor imaging sequences. A less neuroinflammatory environment was observed in IDO^−/−^ mice, as described by reduced microglial activation in the white matter tract, in the cortex, in the hippocampus, and in the caudoputamen. Furthermore, IDO^−/−^ mice showed a reduction in axonal membrane disruption as reported by lower neurofilament light release. The protective effect of indoleamine 2,3-dioxygenase deletion was also associated with attenuation of cardiac arrest-induced abnormality in water diffusion detected with brain magnetic resonance imaging at 24 h after resuscitation. Finally, administration of exogenous l-kynurenine in IDO^−/−^ mice abrogated the protective effects of indoleamine 2,3-dioxygenase deletion on ischemic brain edema and on survival after cardiac arrest. Taken together, these observations suggest that inhibition of the kynurenine pathway after cardiac arrest could represent an important therapeutic target to improve neurologic outcome after resuscitation.

Among different neurobiological cascades implicated in the pathophysiology of post–cardiac arrest brain injury, the role of kynurenine pathway activation has recently emerged^5–7,11^: (1) the kynurenine pathway is activated early after experimental cardiac arrest, and its metabolites increased from 2 h after resuscitation up to 3 to 5 days after cardiac arrest^11^; (2) higher levels of kynurenine pathway metabolites are associated with ICU mortality and 12-month poor neurologic outcome in patients after cardiac arrest^5^; and (3) inhibition of the kynurenine pathway improves neurologic recovery while reducing the pathway activation in both plasma and hippocampus in a rat model of cardiac arrest.^7^ In addition, kynurenine pathway has also been recognized as a key player in the mechanisms and severity of neuronal damage in several other conditions, *i.e.*, traumatic brain injury,^25^ ischemic stroke,^26^ and overall brain dysfunction in ICU patients.^27^

In the current study, a marked improvement in survival and neurologic function was observed in indoleamine 2,3-dioxygenase–deleted mice after experimental cardiac arrest, compared to wild-type animals. The neuroprotective effect of kynurenine pathway inhibition is supported by a comprehensive approach spreading from neurobehavioral assessment to highly translational brain imaging techniques both in short-term (24 h) and long-term (7 days) observations.

Earlier studies suggest that IDO^−/−^ mice do not show any behavioral abnormalities under normal conditions.^28^ Indeed, IDO^−/−^ and wild-type mice showed preservation of locomotor activity and circadian rhythm in physiologic conditions.^28^ In our study, wild-type and IDO^−/−^ animals without cardiac arrest did not show differences in locomotor function. Furthermore, neurologic outcome and locomotor activity appear to be similar between male and female animals without cardiac arrest. A previous report^29^ showed a significant decline in locomotor function after 8 min of cardiac arrest in mice. After cardiac arrest, indoleamine 2,3-dioxygenase–deleted mice showed a higher spontaneous locomotor activity and a significant increase in the total distance traveled compared to wild-type animals. After cardiac arrest, IDO^−/−^ mice also exhibited a maintained cyclical variation in locomotor function, scoring lower during the light phases and higher during the dark phases, as physiologically expected.^30^ A disruption of the circadian rhythms was instead observed in wild-type animals after cardiac arrest, in which the cyclic differences after a light-dark dependent pattern were not shown.

In the diffusion tensor imaging analysis conducted, we showed a higher value of fractional anisotropy in the IDO^−/−^ group, associated with a reduction of the radial diffusivity in the external capsule. Diffusion tensor imaging is emerging as a promising prognostic tool in comatose cardiac arrest patients.^31^ Specifically, the quantification of white matter fractional anisotropy derived from diffusion tensor imaging has been demonstrated to predict outcome with a high degree of accuracy in patients with altered consciousness at day 7 after cardiac arrest, with a lower value of fractional anisotropy associated with unfavorable neurologic outcome.^31^ These results suggest that both a reduction in axonal degeneration and maintenance of myelin structure could be responsible for the preservation of white matter microintegrity observed in IDO^−/−^ mice. To corroborate these findings, lower levels of the biomarker of axonal injury neurofilament light chain were observed in indoleamine 2,3-dioxygenase–deleted mice 7 days after resuscitation.

The crucial role of the kynurenine pathway on neuroinflammation has been previously reported as another pathophysiological mechanism of brain injury related to the activation of this pathway.^32^ In our study, we observed that indoleamine 2,3-dioxygenase deficiency mitigated neuroinflammation at 7 days after cardiac arrest, as represented by a consistent reduction of microglial activation in the white matter tract, in the cortex, in the hippocampus, and in the caudoputamen. We then analyzed the shape parameters of IBA1-positive cells. The perimeter is expected to be higher in activated and ramified cells, and area is expected to increase with hypertrophism due to activation, soma enlargement, and sprouting of new ramifications. We did not detect any significant reduction in the glial fibrillary acidic protein immunostained areas with indoleamine 2,3-dioxygenase deficiency within the white matter tract and the caudoputamen after cardiac arrest. The lack of a significant difference in astrogliosis observed in this study might be due to the model itself. Indeed, the cardiac arrest model induces a global lesion, not a focal lesion, around which a gliotic scar could be formed by activated astrocytes. However, axonal membrane disruption was highlighted in the current study by the elevation in serum levels of neurofilament light chain observed in wild-type animals compared to IDO^−/−^ at 7 days after resuscitation.

Neurofilament light chain protein is a neuronal cytoskeletal intermediate filament protein, essential for cellular integrity and axonal transport. The release of this protein at first in the interstitial fluid, then in the cerebrospinal fluid, and ultimately in the blood occur in the context of neuronal injury and disruption of axonal membrane. Neurofilament light chain recently emerged as a highly accurate biomarker of neuronal injury in predicting neurologic outcome in patients with post–cardiac arrest brain injury.^33^

In the current study, wild-type mice that were successfully resuscitated from 8 min of cardiac arrest exhibited a marked abnormality in water diffusion in the cortex, caudoputamen, and hippocampus 24 h after CPR. The presence of restricted diffusion, corresponding to a low apparent diffusion coefficient value, in the vulnerable regions of the brain strongly correlated with neurologic function. These observations are consistent with clinical studies showing that diffuse abnormalities in diffusion-weighted imaging are associated with poor outcomes in patients resuscitated from cardiac arrest.^34^ In contrast, indoleamine 2,3-dioxygenase deletion markedly attenuated the development of abnormalities in water diffusion in the brain and improved neurologic outcomes. Consistent with clinical findings, in this study, the apparent diffusion coefficient value positively correlates with neurologic function score.

Restricted water diffusion indicates the presence of brain edema presumably resulting from disruption of ion pump function and membrane failure.^35^ The current observations therefore suggest that indoleamine 2,3-dioxygenase deletion can preserve ion pump homeostasis and membrane integrity early after cardiac arrest. Also, a higher activation of the pathway, as represented by a higher kynurenine/tryptophan ratio, is associated with poor neurologic function after cardiac arrest. This result is in accordance with previous studies showing that l-kynurenine administration exacerbated acute neuronal damage after experimental stroke.^36^

The findings of the current study should be interpreted in light of the following limitations. The main limitation is represented by the absence of a group with pharmacologic inhibition of the kynurenine pathway after cardiac arrest. The pharmacologic inhibition of the kynurenine pathway after resuscitation would add clinical relevance to the current findings. Different indoleamine 2,3-dioxygenase inhibitors have been developed over the last years.^37^ In preclinical settings, the most used indoleamine 2,3-dioxygenase inhibitor is represented by 1-methyl-tryptophan. In clinical settings, various compounds have been studied; in the field of oncology, they have been used as immunometabolic adjuvants since 2008;^38^ and phase I and II clinical trials are still ongoing (https://clinicaltrials.gov/ct2/results?cond=ido+inhibitors&term=&cntry=&state=&city=&dist=). Furthermore, we observed significant difference in survival between wild-type and IDO^−/−^ mice at the inspection of the Kaplan–Meier analysis. However, the estimated difference in mortality to calculate the sample size based on a hazard ratio of 0.25 (IDO^−/−^ *versus* wild-type) was mildly overestimated as our data showed a hazard ratio of 0.36.

In conclusion, the current study revealed protective effects of indoleamine 2,3-dioxygenase deletion on neurologic and survival outcomes after cardiac arrest in mice. Our observations suggest that inhibition of the kynurenine pathway could represent a novel target to improve neurologic outcomes after resuscitation.

### Acknowledgments

The authors thank Francesco Casola, Ph.D., Capital Fund Management, Paris, France.

### Research Support

Supported by the ZOLL Foundation (Chelmsford, Massachusetts; to Dr. Magliocca), the Levi−Montalcini Biomedical Sciences Award from the European Society of Intensive Care Medicine (Brussels, Belgium; to Dr. Magliocca), and the Young Investigator Start-up Grant from the European Society of Anesthesiology and Intensive Care (to Dr. Magliocca). The study was also partially funded by Italian Ministry of Health (Rome, Italy), current research IRCCS.

### Competing Interests

Dr. Skrifvars received a fee as speaker from Bard Medical (Ireland). Dr. Ichinose was supported by the National Institutes of Health (Bethesda, Maryland) and was a consultant to Nihon Kohden Innovation Center (Cambridge, Massachusetts), received sponsored research agreement from Cyclerion Therapeutics (Boston, Massachusetts), and serves as an unpaid member of Board of Directors of the ZOLL Foundation. The other authors declare no competing interests.

## Supplemental Digital Content

Supplementary Figure 1. Spontaneous locomotor activity after resuscitation, https://links.lww.com/ALN/D241

Supplementary Figure 2. Neurologic function score without cardiac arrest, https://links.lww.com/ALN/D242

Supplementary Figure 3. Locomotor activity without cardiac arrest, https://links.lww.com/ALN/D243

Supplementary Figure 4. Neurologic function score and kynurenine pathway, https://links.lww.com/ALN/D244

Supplementary Figure 5. Diffusion-weighted imaging after cardiac arrest, https://links.lww.com/ALN/D245

Supplementary Figure 6. Diffusion-weighted imaging without cardiac arrest, https://links.lww.com/ALN/D246

Supplementary Figure 7. Kynurenine Pathway metabolites after l-kynurenine, https://links.lww.com/ALN/D247

Supplementary Figure 8. Rate of return of spontaneous circulation, https://links.lww.com/ALN/D248

Supplementary Figure 9. Fluoro-Jade positive cells after cardiac arrest, https://links.lww.com/ALN/D249

Supplementary Figure 10. neurologic function score in male and female, https://links.lww.com/ALN/D250

Supplementary Figure 11. Spontaneous locomotor activity in male and female, https://links.lww.com/ALN/D251

Supplementary Table. CPR characteristics and hemodynamics, https://links.lww.com/ALN/D252

## Supplementary Material


