# Supplementary material for: Indoleamine 2,3-Dioxygenase Deletion to Modulate Kynurenine Pathway and to Prevent Brain Injury after Cardiac Arrest in Mice
Source: Anesthesiology. 2023 Jul 24;139(5):628–45. doi: 10.1097/ALN.0000000000004713 (PMC10566599; doi:10.1097/ALN.0000000000004713)
Supplement: Supplementary file 1 [file aln-139-628-s001.pdf]

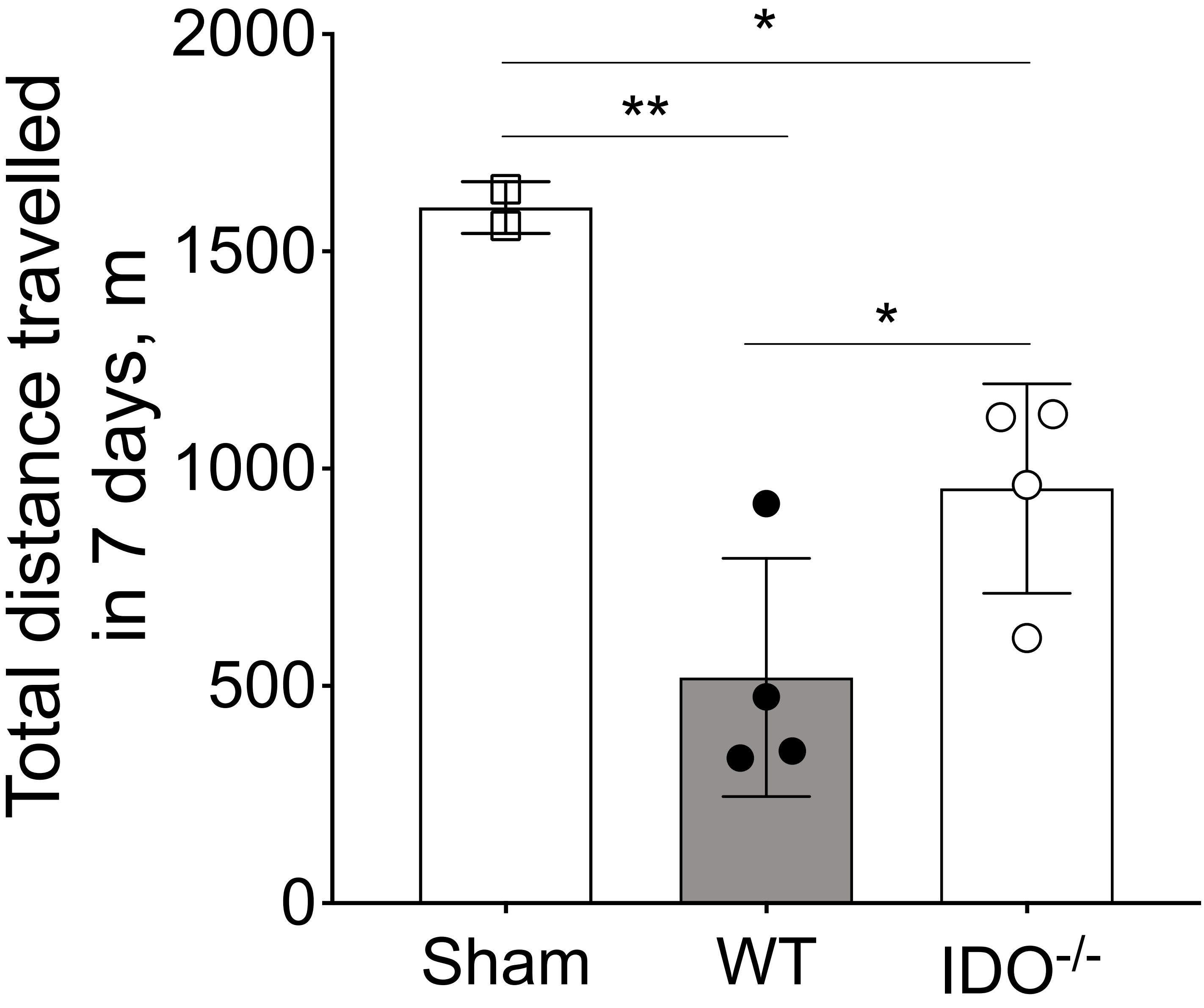
Total distance travelled
in 7 days, m
2000
1500
1000
500
0
*
**
*
Sham
WT
IDO-/-

p=0.0038

Supplementary Figure 1. Spontaneous locomotor activity as total distance travelled by animals during the 7 days following cardiac arrest in WT mice, IDO-/- mice and sham operated mice. Difference between the three study groups was evaluated with using a one-way analysis of variance (1-way ANOVA). Only in the presence of a significant 1-way ANOVA, post-hoc multiple comparisons between groups at different time-points was performed by controlling the false discovery rate using a two-stage step-up method of Benjamini, Krieger and Yekutieli. Treatment effect was reported, $p=0.0038$. *$P<0.05$, **$P<0.01$
