# Supplementary material for: Indoleamine 2,3-Dioxygenase Deletion to Modulate Kynurenine Pathway and to Prevent Brain Injury after Cardiac Arrest in Mice
Source: Anesthesiology. 2023 Jul 24;139(5):628–45. doi: 10.1097/ALN.0000000000004713 (PMC10566599; doi:10.1097/ALN.0000000000004713)
Supplement: Supplementary file 2 [file aln-139-628-s002.pdf]

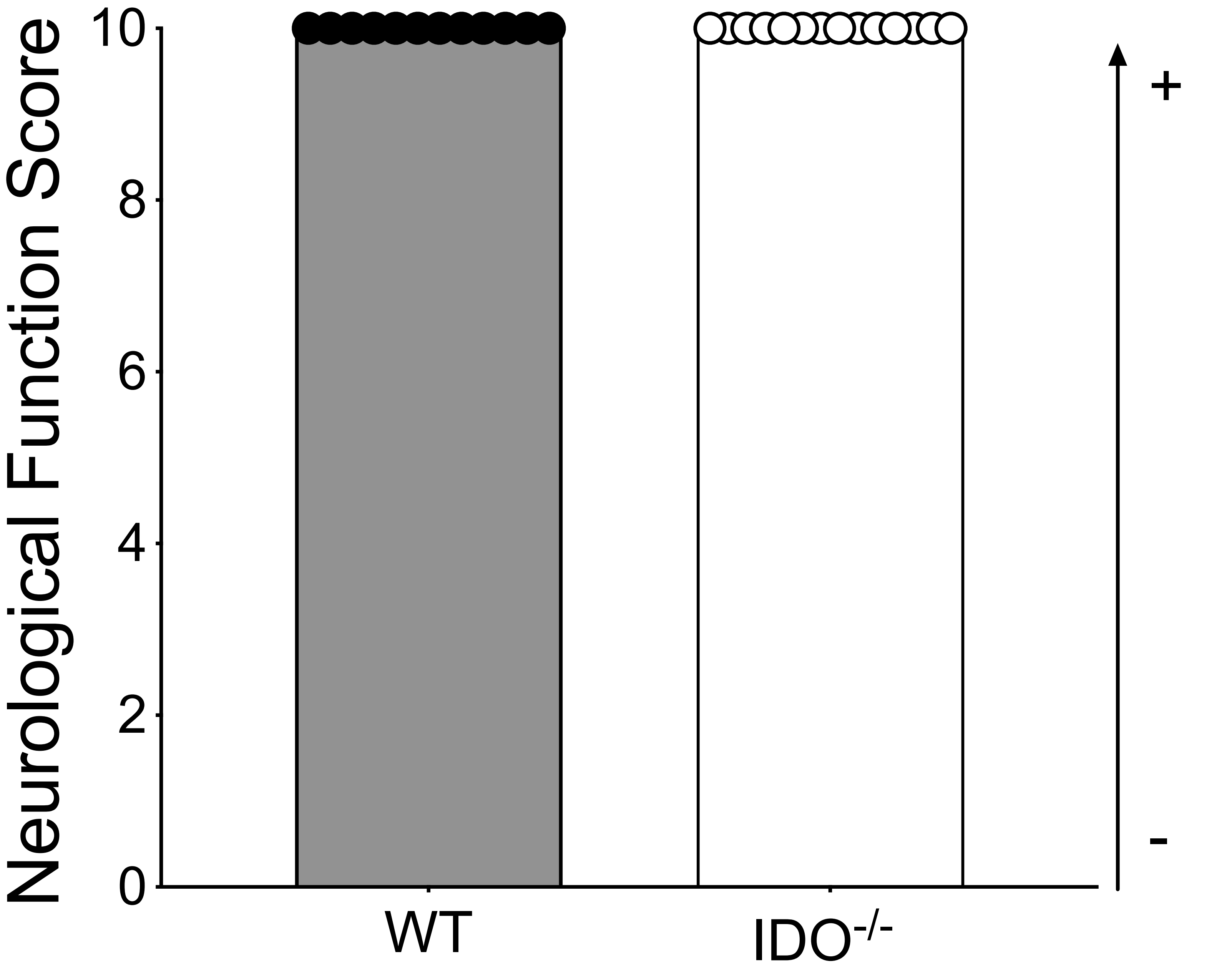
Neurological Function Score
10
8
6
4
2
0
+
-
WT
IDO-/-

Supplementary Figure 2. Neurological function score in WT and IDO-/- animals without cardiac arrest. WT mice (n=12), IDO-/- mice (n=14). All animals scored 10.
