# Supplementary material for: Indoleamine 2,3-Dioxygenase Deletion to Modulate Kynurenine Pathway and to Prevent Brain Injury after Cardiac Arrest in Mice
Source: Anesthesiology. 2023 Jul 24;139(5):628–45. doi: 10.1097/ALN.0000000000004713 (PMC10566599; doi:10.1097/ALN.0000000000004713)
Supplement: Supplementary file 3 [file aln-139-628-s003.pdf]

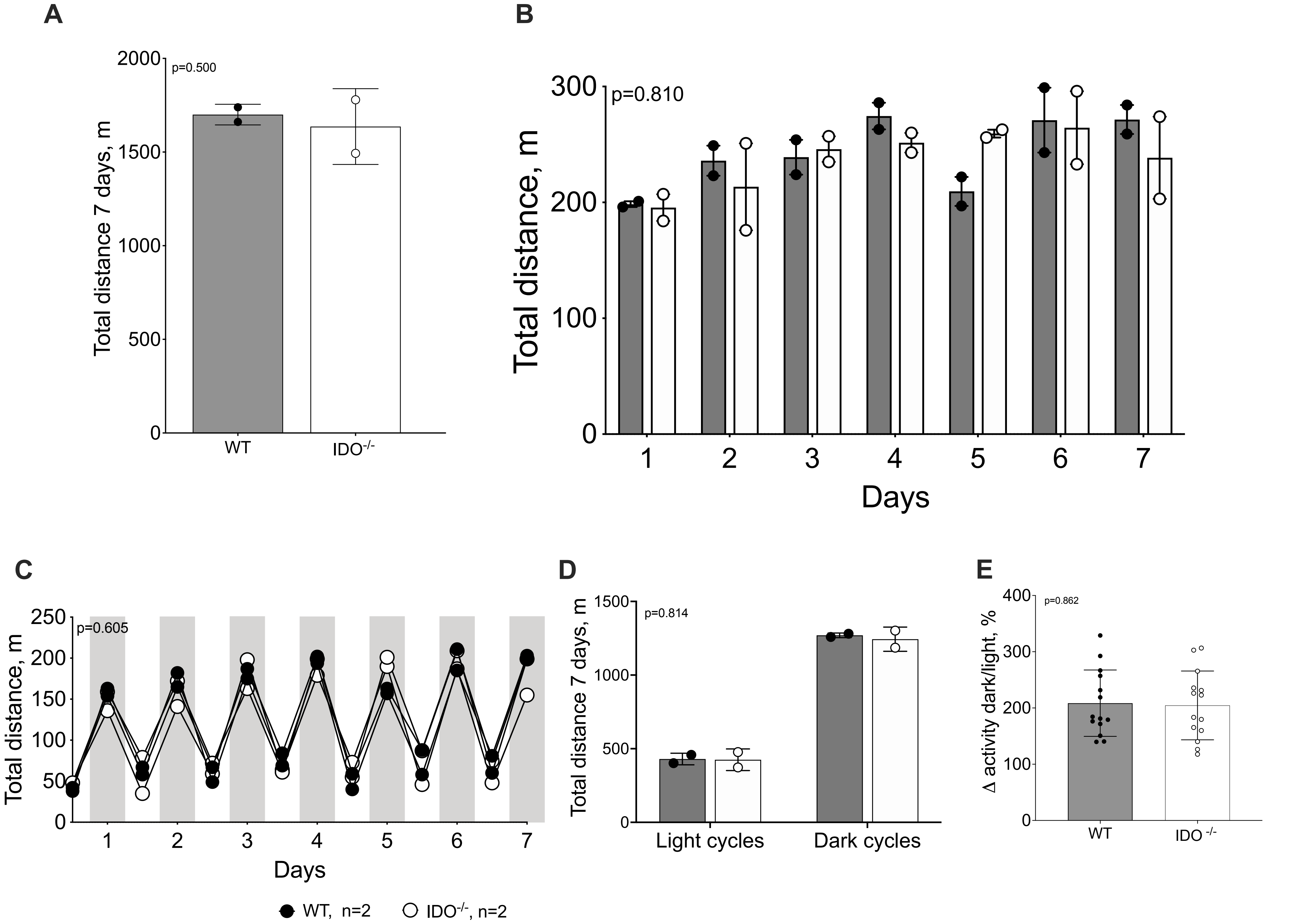

A
p=0.500
Total distance 7 days, m
2000
1500
1000
500
0
WT
IDO-/-
B
p=0.810
Total distance, m
300
200
100
0
1
2
3
4
5
6
7
Days
C
p=0.605
Total distance, m
250
200
150
100
50
0
1
2
3
4
5
6
7
Days
WT, n=2
IDO-/-, n=2
D
p=0.814
Total distance 7 days, m
1500
1000
500
0
Light cycles
Dark cycles
E
p=0.862
Δ activity dark/light, %
400
300
200
100
0
WT
IDO -/-

Supplementary Figure 3. Spontaneous locomotor activity in WT and IDO-/- mice without cardiac arrest. A) Total distance travelled during 7 days. Treatment effect p-value of the mixed effects models for repeated measurement analysis was reported. n=2 animals per group. B) Total distance travelled daily. C) Distance covered daily during the light and dark phases by WT and IDO-/- mice. D) Total distance covered during the light and dark phases by WT and IDO-/- mice. Treatment effect p-value of the mixed effects models for repeated measurement analysis was reported. E) Variation of locomotor activity during the dark/light cycles, expressed as a percentage: (Activitydark-Activitylight)/Activitylight*100).
