# Supplementary material for: Indoleamine 2,3-Dioxygenase Deletion to Modulate Kynurenine Pathway and to Prevent Brain Injury after Cardiac Arrest in Mice
Source: Anesthesiology. 2023 Jul 24;139(5):628–45. doi: 10.1097/ALN.0000000000004713 (PMC10566599; doi:10.1097/ALN.0000000000004713)
Supplement: Supplementary file 4 [file aln-139-628-s004.pdf]

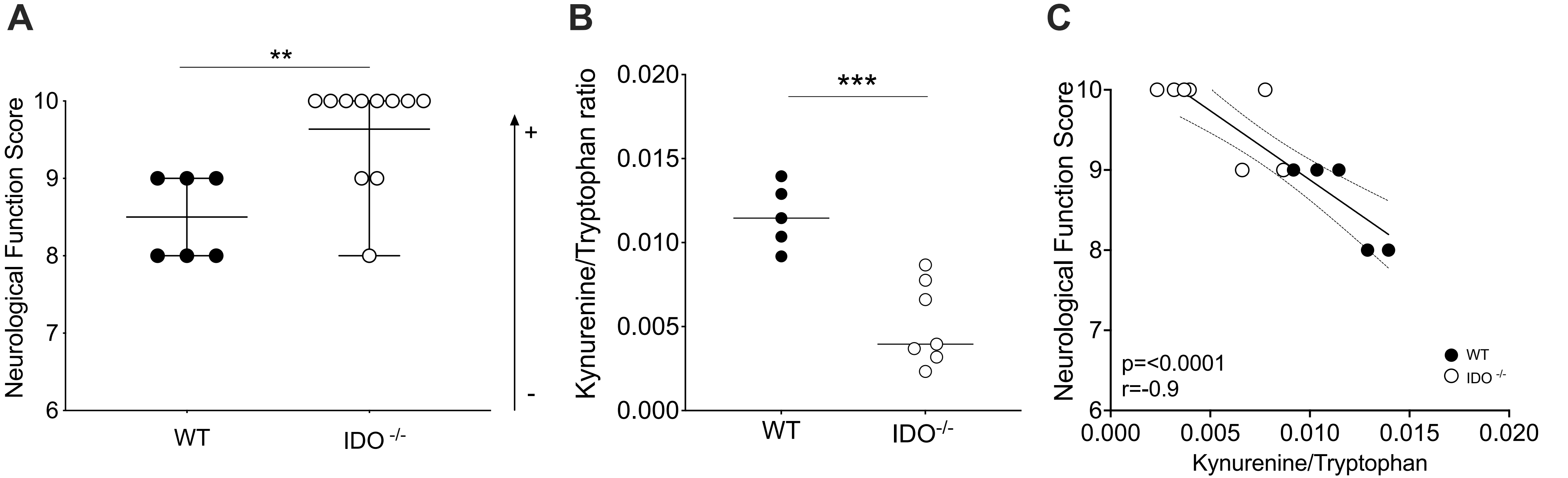

A
**
Neurological Function Score
10
9
8
7
6
WT
IDO -/-
+
-
B
***
Kynurenine/Tryptophan ratio
0.020
0.015
0.010
0.005
0.000
WT
IDO-/-
C
Neurological Function Score
10
9
8
7
6
p=<0.0001
r=-0.9
WT
IDO -/-
0.000
0.005
0.010
0.015
0.020
Kynurenine/Tryptophan

Supplementary Figure 4. Neurological function score in surviving mice (A), kynurenine/tryptophan ratio (B) at 7 days after cardiac arrest. C) Correlation between levels of kynurenine/tryptophan ratio and neurological function score at 7 days after cardiac arrest in the 2 groups. WT mice (n=5-6), IDO-/- mice (n=7-11). Mann-Whitney U test or unpaired Student's t-test was performed according to data distribution. *P<0.05, **P<0.01, **P<0.001.
