# Supplementary material for: Indoleamine 2,3-Dioxygenase Deletion to Modulate Kynurenine Pathway and to Prevent Brain Injury after Cardiac Arrest in Mice
Source: Anesthesiology. 2023 Jul 24;139(5):628–45. doi: 10.1097/ALN.0000000000004713 (PMC10566599; doi:10.1097/ALN.0000000000004713)
Supplement: Supplementary file 5 [file aln-139-628-s005.pdf]

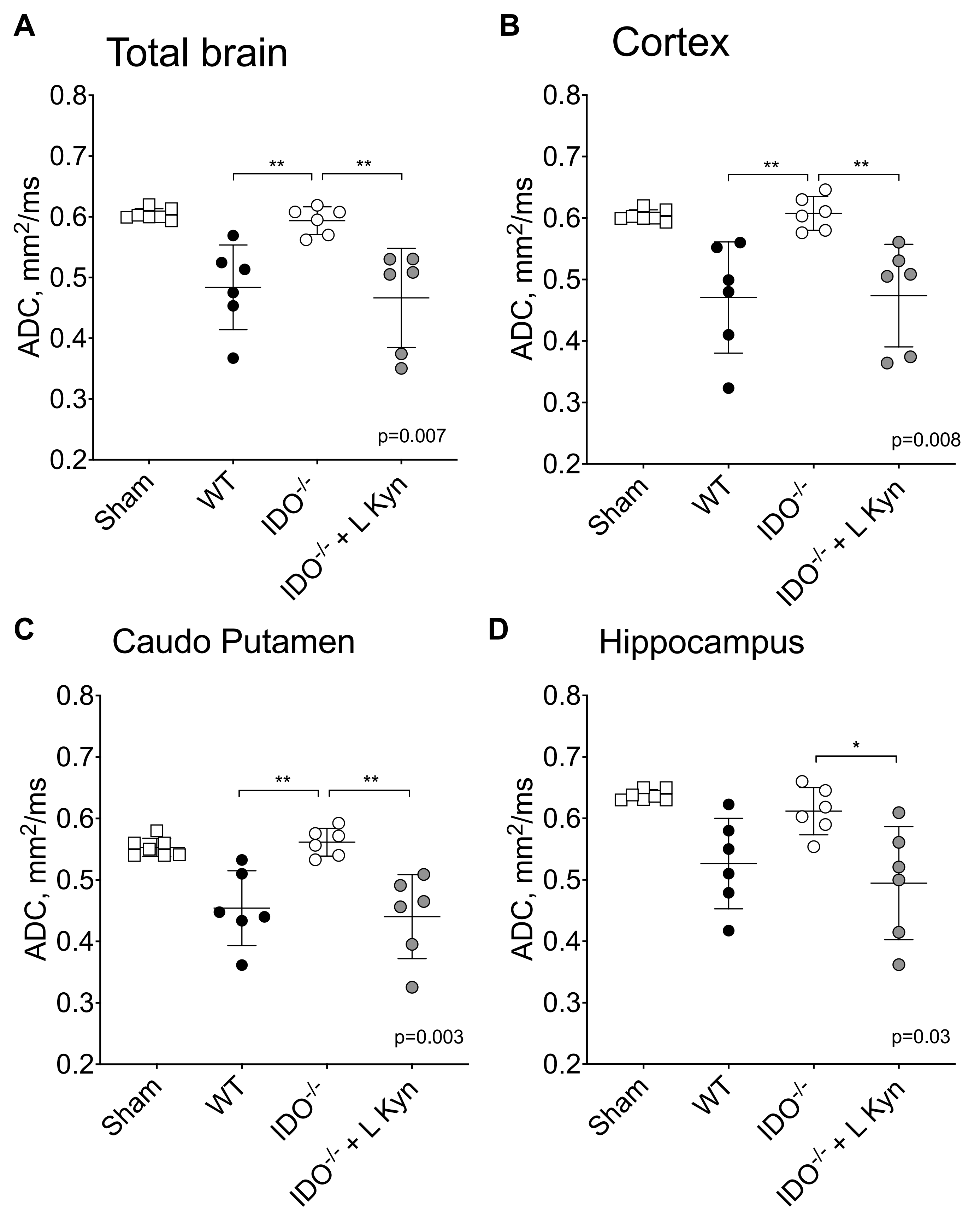

A
Total brain
B
Cortex
C
Caudo Putamen
D
Hippocampus
ADC, mm2/ms
0.8
0.7
0.6
0.5
0.4
0.3
0.2
**
*
p=0.007
p=0.008
p=0.003
p=0.03
Sham
WT
IDO-/-
IDO-/- + L Kyn

Supplementary Figure 5. Apparent diffusion coefficient (ADC) values measured from diffusion-weighted imaging (DWI) sequences acquired 24 hours after cardiac arrest in sham-operated mice, WT mice, IDO-/- mice and IDO-/-+L Kyn mice. (n=6-7 animals per group). A) ADC of the total brain, and in each region of interest: cortex B), caudoputamen C) and hippocampus D) (n=6-7 animals per group). Sham operated mice were not included in the statistical analysis. Difference between the three study groups was evaluated with using a one-way analysis of variance (1-way ANOVA). In the presence of a significant 1-way ANOVA, post-hoc multiple comparisons between groups was performed by controlling the false discovery rate using a two-stage step-up method of Benjamini, Krieger and Yekutieli. *P<0.05, **P<0.01. ADC indicates Apparent Diffusion Coefficient. WT indicates wild-type mice; IDO-/- indicates knock-out mice for Indoleamine 2,3-deoxygenase (IDO). L Kyn indicates L kynurenine.
