# Supplementary material for: Indoleamine 2,3-Dioxygenase Deletion to Modulate Kynurenine Pathway and to Prevent Brain Injury after Cardiac Arrest in Mice
Source: Anesthesiology. 2023 Jul 24;139(5):628–45. doi: 10.1097/ALN.0000000000004713 (PMC10566599; doi:10.1097/ALN.0000000000004713)
Supplement: Supplementary file 6 [file aln-139-628-s006.pdf]

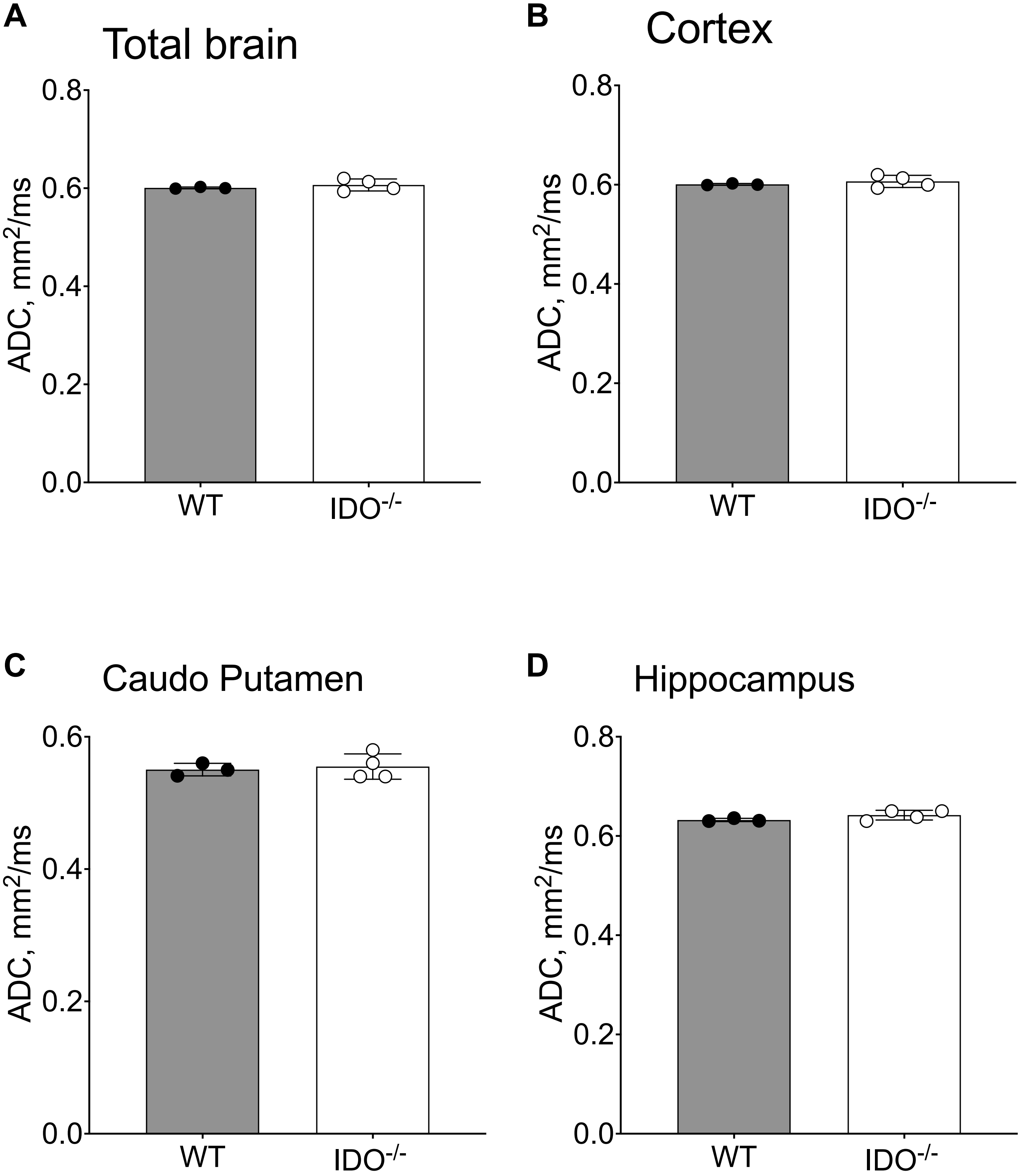
A
Total brain
B
Cortex
C
Caudo Putamen
D
Hippocampus
ADC, mm²/ms
0.0
0.2
0.4
0.6
0.8
WT
IDO-/-

**Supplementary Figure 6.** Apparent diffusion coefficient (ADC) values measured from diffusion-weighted imaging (DWI) sequences acquired in sham-operated mice, WT and IDO-/- mice (n=3 animals per group). A) ADC of the total brain, and in each region of interest: cortex B), caudoputamen C) and hippocampus D). Difference between the three study groups was evaluated with using a one-way analysis of variance (1-way ANOVA). In the presence of a significant 1-way ANOVA. WT indicates wild-type mice; IDO-/- indicates knock-out mice for Indoleamine 2,3-deoxygenase (IDO).
