# Supplementary material for: Indoleamine 2,3-Dioxygenase Deletion to Modulate Kynurenine Pathway and to Prevent Brain Injury after Cardiac Arrest in Mice
Source: Anesthesiology. 2023 Jul 24;139(5):628–45. doi: 10.1097/ALN.0000000000004713 (PMC10566599; doi:10.1097/ALN.0000000000004713)
Supplement: Supplementary file 7 [file aln-139-628-s007.pdf]

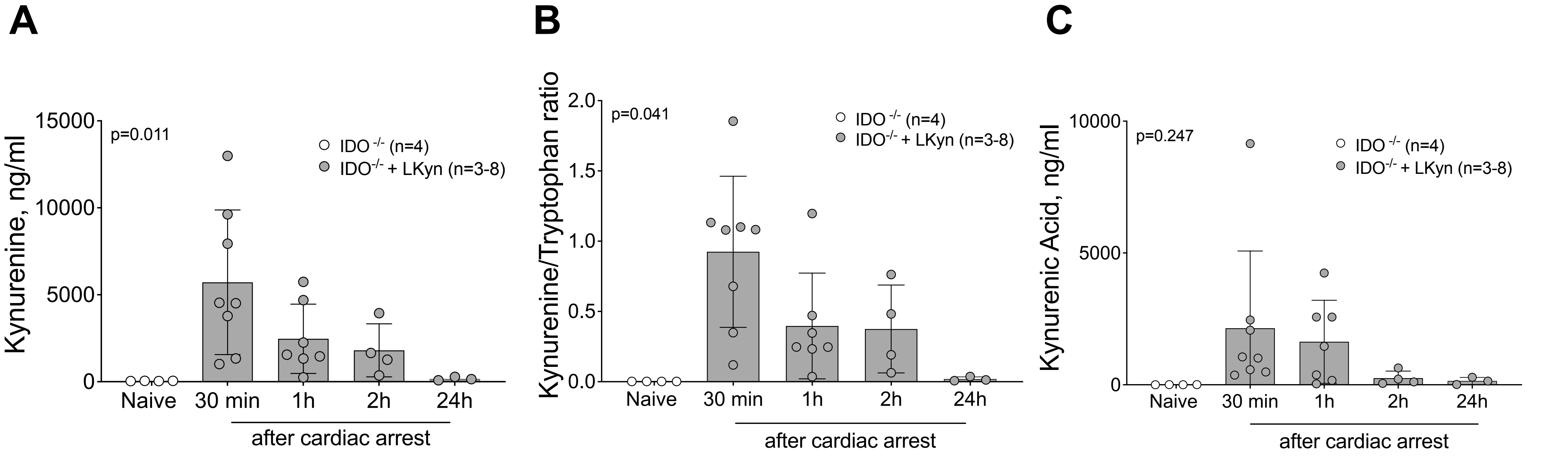

A
p=0.011
IDO -/- (n=4)
IDO-/- + LKyn (n=3-8)
Kynurenine, ng/ml
15000
10000
5000
0
Naive
30 min
1h
2h
24h
after cardiac arrest
B
p=0.041
IDO -/- (n=4)
IDO-/- + LKyn (n=3-8)
Kynurenine/Tryptophan ratio
2.0
1.5
1.0
0.5
0.0
Naive
30 min
1h
2h
24h
after cardiac arrest
C
p=0.247
IDO -/- (n=4)
IDO-/- + LKyn (n=3-8)
Kynurenic Acid, ng/ml
10000
5000
0
Naive
30 min
1h
2h
24h
after cardiac arrest

Supplementary Figure 7. Kynurenine Pathway metabolites concentration in IDO-/- naïve mice and in IDO-/- mice with L Kyn administration at 30 min, 1h, 2h and 24 h after cardiac arrest. A) Kynurenine concentration, B) Kynurenine/Tryptophan ratio and C) Kynurenic Acid concentration in IDO-/- naïve mice and in IDO-/- + L Kyn administration at 30 min, 1h, 2h and 24 h after cardiac arrest is reported. IDO-/- indicates knock-out mice for Indoleamine 2,3-deoxygenase (IDO). L Kyn indicates L kynurenine. Difference between the three study groups was evaluated with using a one-way analysis of variance (1-way ANOVA). In the presence of a significant 1-way ANOVA, post-hoc multiple comparisons between groups was performed by controlling the false discovery rate using a two-stage step-up method of Benjamini, Krieger and Yekutieli.
