# Supplementary material for: Indoleamine 2,3-Dioxygenase Deletion to Modulate Kynurenine Pathway and to Prevent Brain Injury after Cardiac Arrest in Mice
Source: Anesthesiology. 2023 Jul 24;139(5):628–45. doi: 10.1097/ALN.0000000000004713 (PMC10566599; doi:10.1097/ALN.0000000000004713)
Supplement: Supplementary file 8 [file aln-139-628-s008.pdf]

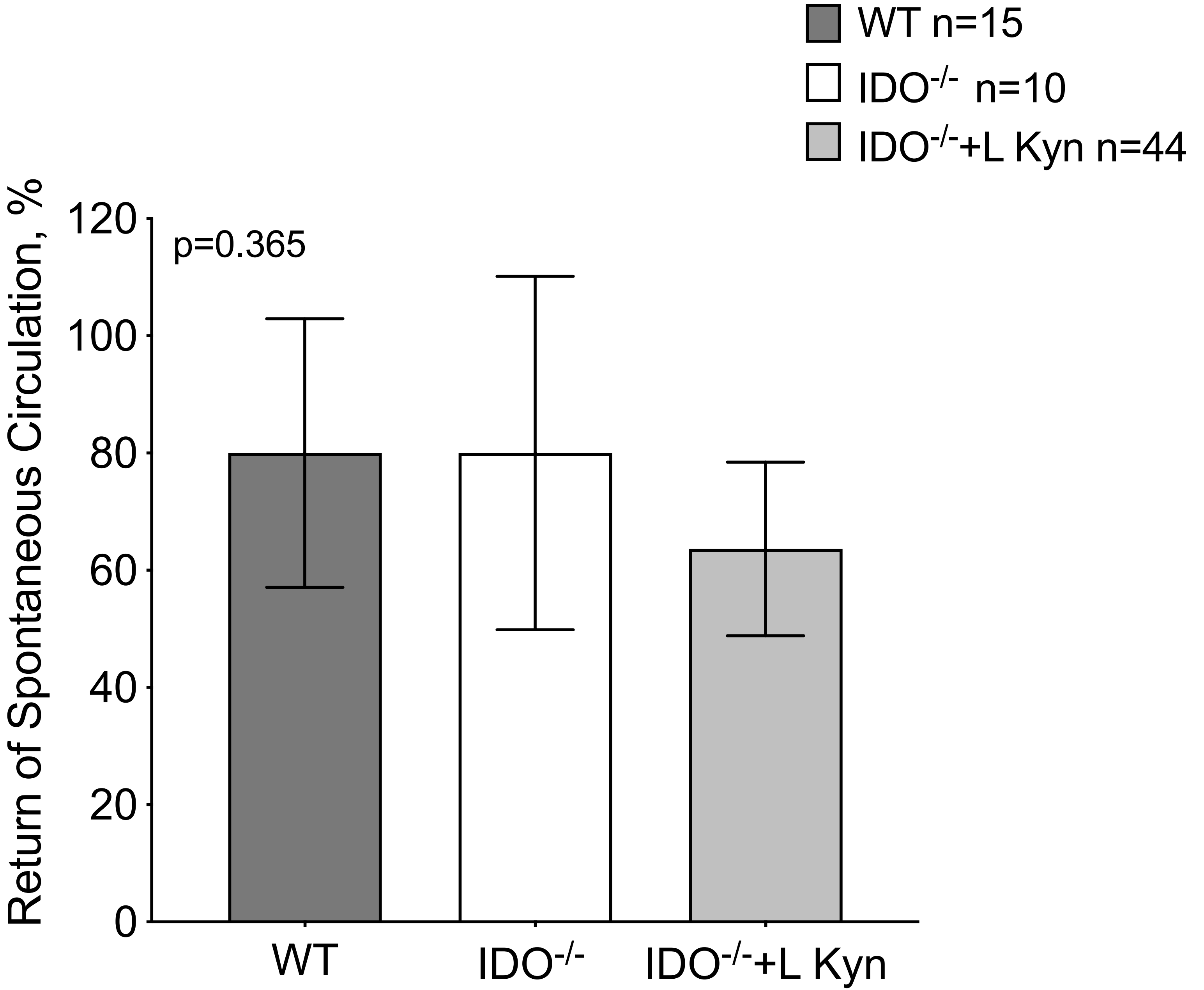
WT n=15
IDO-/- n=10
IDO-/-+L Kyn n=44
Return of Spontaneous Circulation, %
p=0.365
120
100
80
60
40
20
0
WT
IDO-/-
IDO-/-+L Kyn

Supplementary Figure 8. Rate of return of spontaneous circulation in WT (n=15), IDO-/- (n=10) and IDO-/- +LKyn mice (n=44). Chi-square test p=0.365. WT indicates wild-type mice; IDO-/- indicates knock-out mice for Indoleamine 2,3-deoxygenase (IDO); LKyn indicates L kynurenine.
