# Supplementary material for: Indoleamine 2,3-Dioxygenase Deletion to Modulate Kynurenine Pathway and to Prevent Brain Injury after Cardiac Arrest in Mice
Source: Anesthesiology. 2023 Jul 24;139(5):628–45. doi: 10.1097/ALN.0000000000004713 (PMC10566599; doi:10.1097/ALN.0000000000004713)
Supplement: Supplementary file 9 [file aln-139-628-s009.pdf]

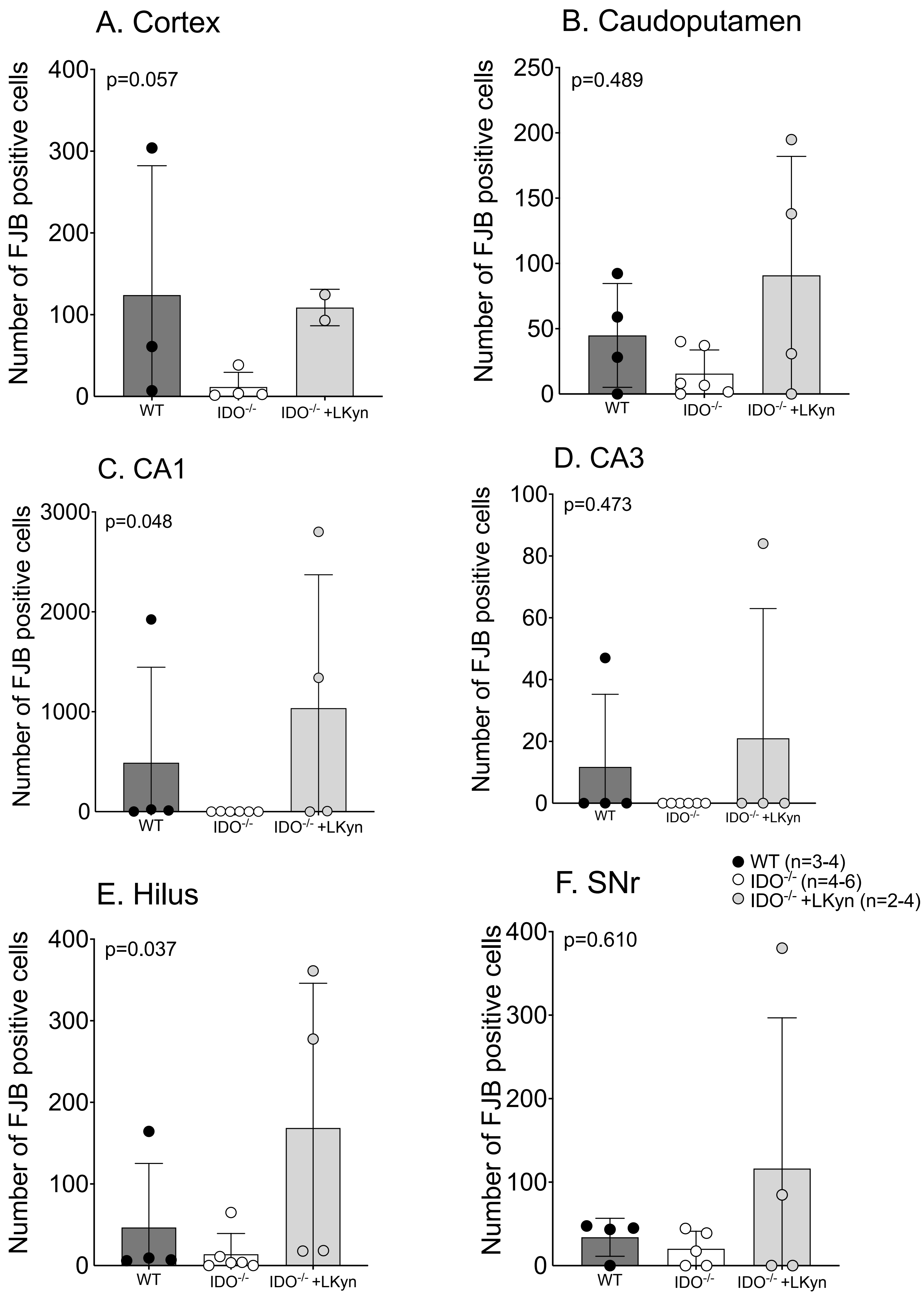
A. Cortex
B. Caudoputamen
C. CA1
D. CA3
E. Hilus
F. SNr
Number of FJB positive cells
p=0.057
p=0.489
p=0.048
p=0.473
p=0.037
p=0.610
WT
IDO-/-
IDO-/- +LKyn
WT (n=3-4)
IDO-/- (n=4-6)
IDO-/- +LKyn (n=2-4)

Supplementary Figure 9. Fluoro-Jade (FJ) positive cells in WT, IDO-/- and IDO-/- +LKyn mice at 24 hours after cardiac arrest. Difference between the three study groups was evaluated with using a one-way analysis of variance (1-way ANOVA) or Kruskall-Wallis according to data distribution. WT indicates wild-type mice; IDO-/- indicates knock-out mice for Indoleamine 2,3-deoxygenase (IDO), L-Kyn indicates L-Kynurenine.
