# Supplementary material for: Indoleamine 2,3-Dioxygenase Deletion to Modulate Kynurenine Pathway and to Prevent Brain Injury after Cardiac Arrest in Mice
Source: Anesthesiology. 2023 Jul 24;139(5):628–45. doi: 10.1097/ALN.0000000000004713 (PMC10566599; doi:10.1097/ALN.0000000000004713)
Supplement: Supplementary file 10 [file aln-139-628-s010.pdf]

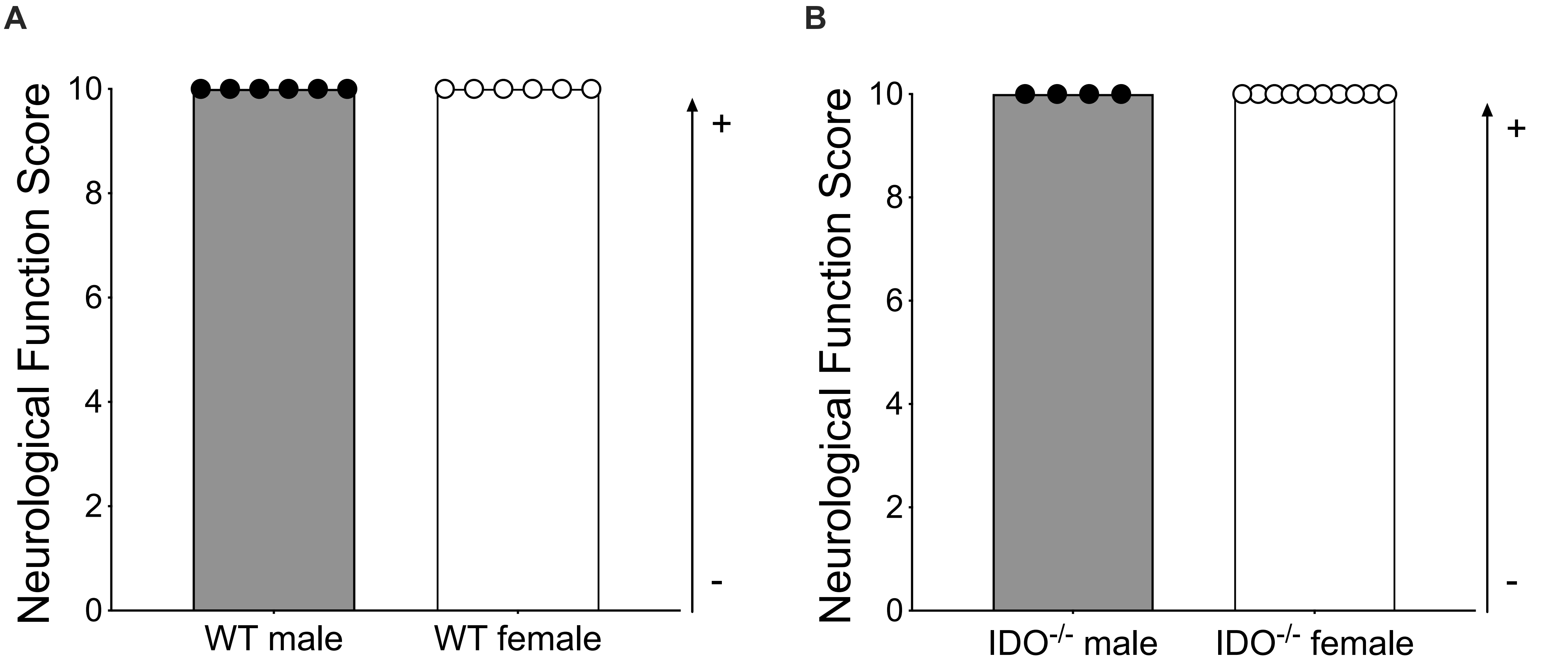
A
B
Neurological Function Score
10
8
6
4
2
0
+
-
WT male
WT female
IDO-/- male
IDO-/- female

Supplementary Figure 10. Neurological function score in male and female WT and IDO-/- animals without cardiac arrest. WT male mice (n=6), WT male mice (n=6), IDO-/- male mice (n=4) IDO-/- female mice (n=10). All animals scored 10.
