# Supplementary material for: Indoleamine 2,3-Dioxygenase Deletion to Modulate Kynurenine Pathway and to Prevent Brain Injury after Cardiac Arrest in Mice
Source: Anesthesiology. 2023 Jul 24;139(5):628–45. doi: 10.1097/ALN.0000000000004713 (PMC10566599; doi:10.1097/ALN.0000000000004713)
Supplement: Supplementary file 11 [file aln-139-628-s011.pdf]

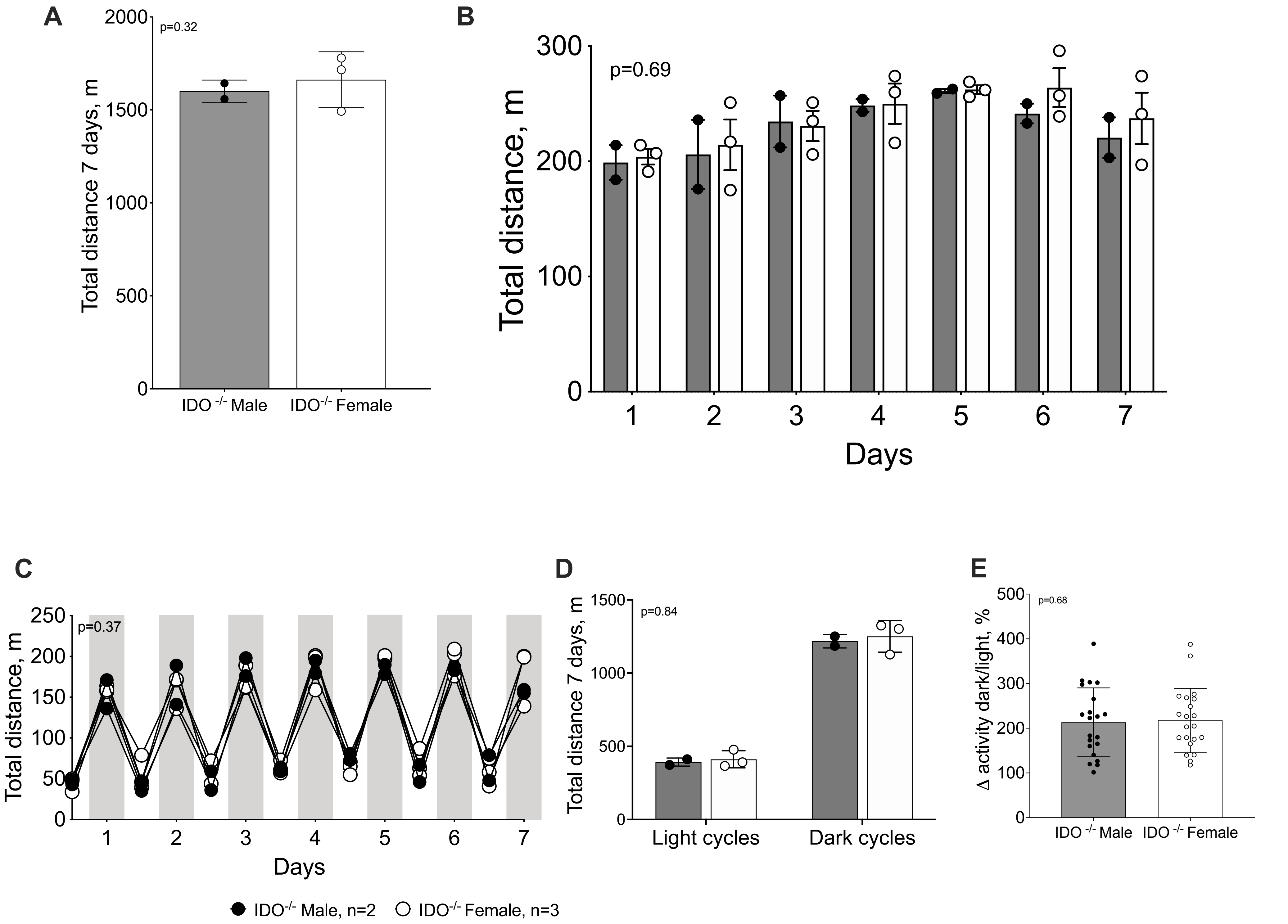
A
p=0.32
Total distance 7 days, m
IDO -/- Male
IDO-/- Female
B
p=0.69
Total distance, m
Days
C
p=0.37
Total distance, m
Days
IDO-/- Male, n=2
IDO-/- Female, n=3
D
p=0.84
Total distance 7 days, m
Light cycles
Dark cycles
E
p=0.68
Δ activity dark/light, %
IDO -/- Male
IDO -/- Female

Supplementary Figure 11. Spontaneous locomotor activity in male and female IDO-/- mice without cardiac arrest. A) Total distance travelled during 7 days. Treatment effect p-value of the mixed effects models for repeated measurement analysis was reported. n=2 animals per group. B) Total distance travelled daily. C) Distance covered daily during the light and dark phases by male and female IDO$^{-/-}$ mice. D) Total distance covered during the light and dark phases by male and female IDO$^{-/-}$ mice. Treatment effect p-value of the mixed effects models for repeated measurement analysis was reported. E) Variation of locomotor activity during the dark/light cycles, expressed as a percentage: (Activitydark-Activitylight)/Activitylight*100).
