# Supplementary material for: Indoleamine 2,3-Dioxygenase Deletion to Modulate Kynurenine Pathway and to Prevent Brain Injury after Cardiac Arrest in Mice
Source: Anesthesiology. 2023 Jul 24;139(5):628–45. doi: 10.1097/ALN.0000000000004713 (PMC10566599; doi:10.1097/ALN.0000000000004713)
Supplement: Supplementary file 12 [file aln-139-628-s012.pdf]

Supplementary Table 1. CPR characteristics and hemodynamics

	WT (n=6)	$IDO^{-/-}$ (n=6)	$IDO^{-/-}$ +L kyn (n=6)	p
Body weight, g	31±6	32±4	30±5	0.998
Total dose of epinephrine, µg	1.0±0.2	1.0±0.1	1.0±0.2	0.990
CPR time to return of spontaneous circulation, sec	68±17	69±11	65±16	0.899
HR, bpm				0.99
HR before cardiac arrest, bpm	458±59	455±42	474±45	-
HR at return of spontaneous circulation, bpm	446±66	436±44	490±31	-
HR Post-Resuscitation 30, bpm	475±68	451±62	452±44	-
HR Post-Resuscitation 60, bpm	443±87	484±38	409±102	-
MAP, mmHg				0.41
MAP before cardiac arrest, mmHg	79±4	82±9	82±10	-
MAP at return of spontaneous circulation, mmHg	112±13	113±8	108±28	-
MAP 30 Post-Resuscitation, mmHg	60±12	76±19	61±19	-
MAP 60 Post-Resuscitation, mmHg	57±16	72±23	47±11	-

Supplemental Table 1. CPR indicates cardiopulmonary resuscitation; HR indicates heart rate; MAP indicates mean arterial pressure; WT indicates wild-type mice; $IDO^{-/-}$ indicates knock-out mice for Indoleamine 2,3-deoxygenase (IDO); L Kyn indicates L kynurenine.

Data are presented as mean ± SD. Treatment effect p-value of the mixed effects models for repeated measurement analysis was reported. Post-hoc multiple comparisons was performed by controlling the false discovery rate using a two-stage step-up method of Benjamini, Krieger and Yekutieli.
